# Core promoterome of barley embryo

**DOI:** 10.1016/j.csbj.2023.12.003

**Published:** 2023-12-05

**Authors:** Simon Pavlu, Sarvesh Nikumbh, Martin Kovacik, Tadaichi An, Boris Lenhard, Hana Simkova, Pavla Navratilova

**Affiliations:** a Institute of Experimental Botany of the Czech Academy of Sciences, Slechtitelu 31, 77900 Olomouc, Czech Republic; b Department of Cell Biology and Genetics, Faculty of Science, Palacky University, Slechtitelu 27, 78371 Olomouc, Czech Republic; c Merck Sharp & Dohme (UK) Limited, 120 Moorgate, London EC2M 6UR, UK; d DNAFORM Precision Gene Technologies, 230–0046 Yokohama, Kanagawa, Japan; e Computational Regulatory Genomics, MRC London Institute of Medical Sciences, London, UK; f Institute of Clinical Sciences, Faculty of Medicine, Imperial College London, Hammersmith Hospital Campus, London, UK

**Keywords:** Core promoter, Cap Analysis of Gene Expression, Morex, Initiator, TOR-signaling, Hordeum vulgare, Transcription regulation

## Abstract

Precise localization and dissection of gene promoters are key to understanding transcriptional gene regulation and to successful bioengineering applications. The core RNA polymerase II initiation machinery is highly conserved among eukaryotes, leading to a general expectation of equivalent underlying mechanisms. Still, less is known about promoters in the plant kingdom. In this study, we employed cap analysis of gene expression (CAGE) at three embryonic developmental stages in barley to accurately map, annotate, and quantify transcription initiation events. Unsupervised discovery of de novo sequence clusters grouped promoters based on characteristic initiator and position-specific core-promoter motifs. This grouping was complemented by the annotation of transcription factor binding site (TFBS) motifs. Integration with genome-wide epigenomic data sets and gene ontology (GO) enrichment analysis further delineated the chromatin environments and functional roles of genes associated with distinct promoter categories. The TATA-box presence governs all features explored, supporting the general model of two separate genomic regulatory environments. We describe the extent and implications of alternative transcription initiation events, including those that are specific to developmental stages, which can affect the protein sequence or the presence of regions that regulate translation. The generated promoterome dataset provides a valuable genomic resource for enhancing the functional annotation of the barley genome. It also offers insights into the transcriptional regulation of individual genes and presents opportunities for the informed manipulation of promoter architecture, with the aim of enhancing traits of agronomic importance.

## Highlights

• Barley core promoter architecture types related to GO terms and epigenetic profiles were postulated.
• Previously undescribed alternative promoters and promoter shifts between the analyzed embryo stages were revealed.
• CAGE data provided reliable support for improving gene annotation in the barley reference genome.
• A comprehensive barley embryo promoterome dataset is provided for community use.
• New, more complete GO annotation for the reference barley gene set was generated.

## Graphical Abstract

**Figure:**
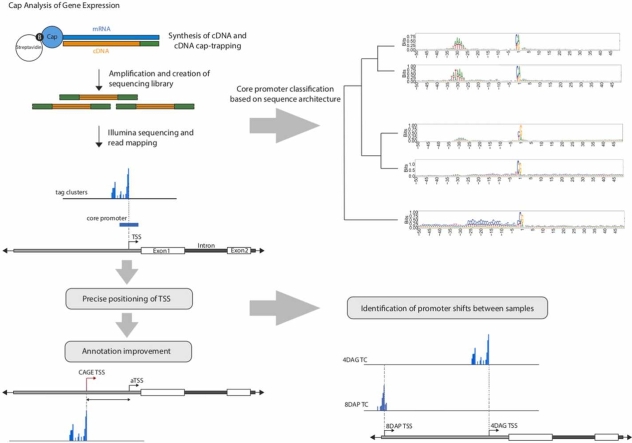

## 1 Introduction

Biotechnological research aims to enhance plant utility and achieve greater resilience by exerting complete control over gene expression at two fundamental levels. Firstly, control of transcription is achieved by regulating the quantity of mRNA produced from a specific gene. The second level involves post-transcriptional events that govern the translation of mRNA into proteins. A comprehensive understanding of these processes is gained by elucidating the function of each nucleotide within the specific genomic sequences involved in controlling expression. The core promoter serves as the ultimate platform for integrating signals of transcriptional regulation, and the complete set of these core promoters in a given species is referred to as the 'promoterome'. The core promoter of eukaryotic RNA polymerase II is defined as the minimal sequence at the 5′ end of a gene, essential for initiating transcription. This sequence is recognized and bound by general transcription factors through the TFIID complex, the largest multiprotein entity composed of the TATA-binding protein (TBP) and approximately 12–13 TBP-associated factors (TAFs) [1]. Such binding is a prerequisite for the assembly of the RNA polymerase pre-initiation complex (PIC), leading to the subsequent events that trigger transcription initiation. Therefore, core promoter regions encompass sequences at the 5′ termini of all mRNAs transcribed by RNA polymerase II, starting from the very first nucleotide of the full-length transcript, which are often imprecisely defined in genome annotations. Crucially, promoter diversity stems from varying cofactor binding and activity – a pivotal aspect for modulating gene responsiveness to transcriptional enhancers, the mechanisms of which are not yet fully elucidated in plants [2], [3], [4].

Promoter mechanics and sequence composition have been extensively studied in metazoans, with the aim of categorizing these elements to provide a roadmap for their understanding. This topic has been reviewed comprehensively in [5]). A number of well-defined core promoter sequence elements have been characterized, including the initiator element (Inr), TATA box, TFIIB recognition element (BRE), core promoter motif ten element (MTE), downstream promoter element (DPE), among others, which often co-occur in various combinations (reviewed in [6]). Transcription initiation in humans and Drosophila is known to commence from a well-characterized, extended Initiator sequence (YYANWYY and TCAKTY, respectively), which may even be sufficient for transcription initiation on its own [7]. Despite their significance and evolutionary conservation, new and lineage-specific promoter elements, located both upstream and downstream of the transcription start site (TSS), continue to be identified in plants (e.g., the Y patch [8], [9] or the TC-motif [10]), protozoa and animals [11], [12], [13]. The interplay between these elements and variants of the initiator influences the pre-initiation complex (PIC) composition, TSS selection, level of polymerase engagement, transcriptional burst size, cis-regulatory responsiveness, and polymerase pausing [14], [15]. These factors collectively have direct implications for gene expression regulation. The common initiator sequence, located at positions − 1 and + 1 relative to the TSS, is a pyrimidine-purine (PyPu) dinucleotide, a pattern confirmed in Arabidopsis, maize and rice promoters [8], [9]. The sequence downstream of the initiator, within the 5′ untranslated region (5′UTR), determines the binding affinity for specific TATA-box associated factors (TAFs) and may include the 5′ Terminal OligoPyrimidine (TOP) motif, a sequence responsive to target of rapamycin (TOR) signalling. TOR is a protein kinase conserved across eukaryotes that orchestrates metabolic regulation by promoting anabolic processes in the presence of nutrients. In Arabidopsis, TOR is essential for early embryogenesis and for survival under stressful conditions [16].

Despite the variety of scenarios, each species has a limited number of synergistic motif/transcription factor (TF) combinations, with two primary configurations being highlighted: TATA-Inr-MTE and BREu-Inr-MTE, as supported by a PIC structure study [17]. This classification is consistent with the two principal classes of promoters identified in mammals based on the number and distribution of transcription start sites: focused promoters, characterized by a single initiation site approximately 5 base pairs wide ('sharp' initiation), and dispersed promoters, which feature multiple initiation sites spanning up to 100 base pairs ('broad' initiation) [18]. These classes correlate closely with chromatin configurations, including nucleosome positioning, histone variants [19] and their posttranslational modifications [20], which determine the motif-independent 'PIC catchment area' [21]. Intriguingly, these core promoter configurations are also associated with distinct categories of genes, a pattern that appears to be consistent across many organisms, including plants [9], [22].

The path to characterizing promoters can be taken from gene annotation or promoter prediction methods, however, the direct proof of promoter position and distribution of TSSs is provided by sequencing of long capped RNA species called Cap analysis of gene expression (CAGE) [23] or similar methods, e.g. GRO-seq, Smar2C2, TSS-seq, [9], [24], [25]. CAGE, in particular, has been adopted as a fundamental method by large genome consortium projects, aiding in accurate gene annotation and providing insights into gene expression levels [26], [27]. Compared to metazoans, plant promoter characterization has been the focus of only a few systematic studies, largely confined to Arabidopsis [28], soybean, rice, sorghum, wheat and maize [9], [22]. These studies suggest that sequence features of promoters may vary among plant species. Detailed functional analysis of promoters, using monocot and dicot reporter systems across three species - Arabidopsis, maize, and sorghum - has confirmed species-specific, sequence-dependent variations in promoter activity and strength [29]. This indicates that there are differences in the transcriptional machinery both between and within these two groups. The authors have experimentally demonstrated the effects of promoter mutations, highlighting the significance of informed promoter design in transgenesis.

Considering the vital importance of Triticeae crops—wheat, barley, and rye—in human and animal diets, barley (*Hordeum vulgare* L.) presents itself as a suitable model for real-world studies of promoter architecture due to its status as a diploid species with a reference genome assembled to near telomere-to-telomere (T2T) completion [30], [31] and the availability of established transgenesis protocols [32]. This makes barley a suitable candidate for research with potential applicability to related Triticeae species. In this study, we generated CAGE datasets from three developmental stages of barley embryos in order to identify promoters involved in various differentiation processes and to elucidate the dynamics of transcription start site usage throughout development. Our data uncovered discrepancies between annotated and detected TSSs, as well as the existence of alternative TSSs, which presumably influence both regulatory and protein-coding sequences. The application of a novel core-promoter sequence clustering approach, combined with the examination of gene functions and epigenetic characteristics, indicated a functional divergence among genes associated with different promoter categories, reflecting disparate cofactor dependencies and modes of transcriptional regulation. Overall, we have demonstrated the power of CAGE promoterome profiling across multiple plant developmental stages in providing nucleotide-level positions along with initiation metrics for all potential alternative TSSs of every active gene. The resultant promoterome dataset lays the groundwork for deciphering the logic of transcriptional regulation and offers a valuable genomic resource for research and agricultural biotechnology applications in barley.

## 2 Methods

### 2.1 CAGE

Barley cv. Morex was grown in growth chambers at 16/8 hrs light cycle, 16 °C day/12 °C night temperature. For the 4DAG seedlings, seeds were germinated on wet tissue paper at 20 °C for 4 days before harvesting and removing remnants of seed coat and endosperm. The 8- and 24DAP embryos were staged according to their time of fertilization, size and phenotype and dissected as described previously [33]. Total RNA was extracted by Monarch® Total RNA Miniprep Kit (NEB cat#T2010S) and its quality was checked by Bioanalyzer (Agilent) to ensure that the RIN (RNA integrity number) values were over 7.0. CAGE libraries were sequenced using single-end reads of 150 bp on a NovaSeq instrument (Illumina). CAGE library preparation, sequencing, and read mapping on MorexV3 annotation were performed by DNAFORM (Yokohama, Kanagawa, Japan).

### 2.2 CAGE data and motif analysis

**Fig. 1:**
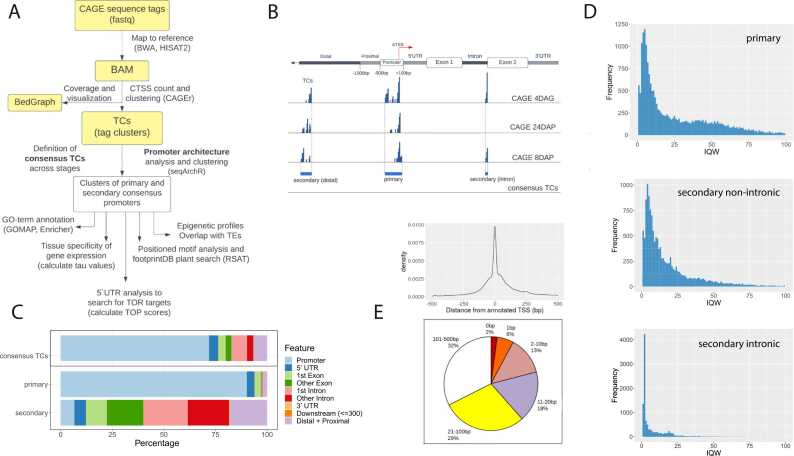
**Initial analysis of the barley CAGE dataset. A**) CAGE data analysis workflow. **B**) Definition of the consensus tag clusters (TCs) and of the primary and the secondary promoter set. The aTSS corresponds to the TSS as in MorexV3 annotation. **C**) Annotation of consensus promoters. The consensus TCs dataset (top bar) was split into the primary and secondary sets (second and third bars) using a filtering method described in the main text. **D)** Distribution of 24DAP promoter interquantile width (IQW) values for primary, secondary non-intronic and secondary intronic TCs. **E**) Distribution of distances between dominant CTSSs and aTSSs for barley genes expressed in 8DAP, 24DAP and 4DAG embryos (top), and proportions of expressed genes with a dominant CTSS located within the designated distance from the aTSS (bottom). The analyses were done for dominant CTSSs whose distance from the aTSS was not greater than 500 bp.

Obtained reads (CAGE tags) were mapped to the MorexV3 genome using BWA (version 0.7.17). Unmapped reads were then mapped by HISAT2 (version 2.0.5) while rRNA reads were filtered. Mapping rates varied between 41% and 87% of total reads. Regions that had a 90% overlap between replicates were extracted by BEDtools (version 2.12.0). Tag count data for each of the samples were clustered using the CAGEr program, which merged neighbouring TSSs with mutual distance < 20 bp into a single tag cluster (TC). Clusters with tags per million (TPM) < 0.1 were discarded just as singletons that had a TPM signal < 5. MorexV3 annotation (both high and low confidence genes) was used to assign a genomic category to each promoter candidate by applying the ‘annotatePeak’ function from the ChipSeeker package [34]. The genomic features were assigned according to the following hierarchy: promoter > 5′UTR/3′UTR > exon > intron > proximal, where ‘promoter’ has been defined as ranging from − 500 to + 100 bp and ‘proximal’ from − 1000 to − 500 bp relative to the annotated TSS (aTSS) of the nearest gene (Fig. 1B). For each gene ID, the hierarchically highest feature was assigned. In case two candidates had the same hierarchical significance, the candidate located closer to the aTSS was taken as the primary promoter. The rest were set aside as the ‘secondary promoter dataset’. For further analysis, the ‘consensus’ clusters were determined using the ‘aggregateTagClusters’ CAGEr function, which aggregates TSSs from the three samples, merging those with mutual distance < 100 bp. The set of consensus clusters created by this method was filtered and split between the primary and secondary TC datasets as described above.

The consensus candidates from the CAGEr were further clustered according to their sequence similarity using a seqArchR program (v1.2.0, Nikumbh 2023). The seqArchR configurations were kept default with the exceptions of ‘bound’ being set to 10^− 8 and ‘chunk size’ to 5000 for primary promoters (500 for the secondary set) to better suit our dataset. The resulting 49 clusters for the primary dataset were further collated into nine final seqArchR clusters based on sequence logo similarity using the seqArchRplus utilities. The 27 secondary clusters were collated into the final seven. Scripts with more details can be found in the github repository.

The + /- 50 bp around all TC`s dominant TSSs were subjected to peak-motif position analysis by RSAT (Regulatory Sequence Analysis Tools) followed by the hierarchical matrix-clustering method to generate a summary radial tree, both under custom settings documented in the rsat_analysis.txt deposited in the GitHub repository. The TOP score was calculated according to [35] from the 4DAG CAGE BAM file. The calculation script is available at: https://github.com/carsonthoreen/tss_tools/blob/master/tss_analyzer.py.

### 2.3 Tissue specificity and GO-term annotation of promoter clusters

The tissue specificity values were calculated by applying the Tau algorithm written as R script (deposited at https://rdrr.io/github/roonysgalbi/tispec/man/) on tpm (transcripts per million) matrix from 18 distinct samples from the EoRNA (https://ics.hutton.ac.uk/eorna/index.html) datasets.

To increase the accuracy of barley GO term annotation we have utilized the Gene Ontology Meta Annotator for Plants (GOMAP)-singularity pipeline [36], which combines three different annotation techniques using the protein fasta file as an input. With the new barley GO-term annotation in hand, the GO-terms were assigned to each gene of the seqArchR clusters. Using the ‘Enricher’ function from the ‘clusterProfiler’ package with default settings we determined the enrichment of GO terms within the clusters, calculating the p-value based on the hypergeometric model. The enriched GO terms together with their p-values were then loaded into the REVIGO web interface [37] to reduce the redundancy of the enriched set and differentiate between the BP (biological process), CC (cellular compartment) and MF (molecular function) GO-term categories. The raw TSV REVIGO data for each of these categories were then used to produce dot plots, showcasing only the top five most enriched GO terms for each of the seqArchR clusters.

### 2.4 Histone modification ChIP-seq and MNase-seq data analysis

Barley embryos grown and collected for the CAGE were used for nuclei isolation and MNase digestion followed by native ChIP-seq as described previously [38], with modifications detailed in [31]. These ChIP-grade antibodies against modified histones were used: H3K4me3 (Abcam ab8580) and H3K27me3 (Diagenode C15410195). Resulting sequencing libraries, including those generated from MNase-digested input, were sequenced on the NovaSeq6000 platform. Reads from the ChIP-seq pipeline went through qualitative trimming by Trim Galore (v. 0.6.2) and mapping to the MorexV3 reference genome was performed using the Bowtie 2 package (version 2.4.2). Duplicated reads were then removed using Picard tools (version 2.9.0) and MACS2 software was used to call peaks. For creating heatmaps, the deeptools functionalities computeMatrix and plotHeatmap were utilized, with the kmeans clustering set to 2, to identify any divergence within the promoter clusters. ChromHMM analysis was performed according to the ChromHMM protocol [39]. MNase-digested input WIG files were used to calculate nucleosome positions around dominant CAGE TSSs (CTSSs) using DANPOS software https://github.com/sklasfeld/DANPOS3. The resulting WIG files were plotted using deeptools` computeMatrix and plotProfile functions.

### 2.5 RNA-seq and CAGE correlation data analysis

The 4DAG [40] and 8/24DAP [41] RNA-seq datasets were analyzed using the RSEM software with STAR mapping pipeline [42], [43]. The CAGEr datasets were constructed using CAGEr, merging replicas and normalizing tag counts to TPM. ChrUn records were filtered out of both CAGE and RNA-seq datasets and only records that had a TPM value higher than 0.1 were considered.

## 3 Results

### 3.1 Cap analysis of gene expression profiles transcription initiation events in barley embryos

To identify transcription initiation events genome-wide in barley cv. Morex, we applied CAGE to total RNA isolated from three embryonic developmental stages: eight days after pollination (8DAP), 24 days after pollination (24DAP) and four days after germination (4DAG), each in two highly correlated replicates each (BAM file Pearson correlation 0.99; Figure S1A). Sequencing of the CAGE samples yielded 52,313,604; 57,927,548; and 60,825,231 CAGE tags from the 8DAP, 24DAP, and 4DAG samples, respectively. Data analysis followed the workflow depicted in Fig. 1A. We employed the CAGEr software [44] to merge replicates, identify CTSSs, and aggregate them into 49,848; 51,903 and 54,276 TCs for 8DAP, 24DAP, and 4DAG, respectively, with custom parameter settings (Figure S1B). Each TC was characterized by its interquantile width (IQW), indicative of promoter breadth, and tag counts, expressed as tags per million, reflecting the expression level. Additionally, each TC was annotated with the position of a dominant CTSS and the associated gene identifier (ID). All TCs were annotated using the ChipSeeker software [34] according to genomic features and gene IDs, considering both high- and low-confidence genes annotated on the MorexV3 pseudomolecules [30] (Figure S1C). TCs located more than 1000 bp from the nearest annotated TSS - a total of 7662; 7296 and 7390 TCs for 8DAP, 24DAP, and 4DAG, respectively - were considered unassociated with the nearest gene and were analyzed as a secondary TC set. We applied a hierarchical approach to retain a single TC, representing the most likely promoter, for each gene (see the Methods section and Fig. 1B for details). This procedure resulted in a primary TC set comprising 19,289; 19,567 and 20,878 promoter candidates for 8DAP, 24DAP, and 4DAG, respectively. The remaining TCs were allocated to the secondary TC set, which included alternative promoters as well as intragenic and intergenic TSSs, the latter likely encompassing promoters of presumed unannotated genes.

Our objective was to establish a generalized classification of promoter types, therefore we pooled the primary TCs from all three embryonic stages. Overlapping TCs were merged into a set of 34,897 consensus clusters. Following the same filtering and annotation protocols as described above, we derived 21,610 'primary consensus' and 13,287 'secondary consensus' clusters (Fig. 1B, C, and Table S1). These consensus clusters represented the central dataset for the majority of our analyses.

**Fig. 2:**
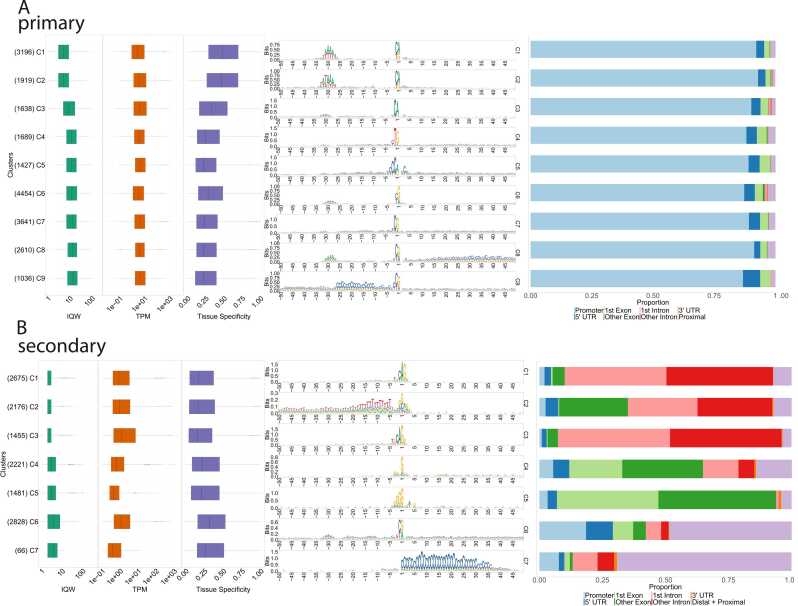
**: Clustering of CAGE promoter sequence architectures.** Clusters of consensus promoters were generated by the seqArchR algorithm and ordered by median IQWs. The composed plots for **A**) primary and **B**) secondary promoter clusters include boxplots for IQW, gene expression level values (tags per million (TPM), log-transformed) and tissue specificity (tau values), followed by sequence logos and genomic feature annotation bar plots. The numbers of genes per cluster are given in parentheses.

A comprehensive catalog of the TCs, which includes details such as the consensus-TC coordinates in the MorexV3 genome, the assigned gene IDs, positions of the dominant TSSs, feature annotations, classifications, IQWs, and TPMs of all TCs in the sense direction, is provided in Data S1. This dataset is also available in the Eukaryotic Promoter Database (EPD) at https://epd.expasy.org/epd/. Given the recognized importance of promoter width in distinguishing different functional classes of promoters, we compared the IQW distributions between the primary and secondary consensus cluster sets. We observed a tendency towards narrower TCs in the secondary set (Figs. 1D and 2). Within the secondary TC set, we identified a subgroup of extremely narrow (width 1–2 bp) TCs, which we termed 'singletons'. These singletons were frequently initiated at the first intron-exon boundary (CAG sequence) and were characterized by notably high TPM values.

### 3.2 CAGE data improve gene annotation and provide transcription initiation levels

Promoters are characterized by distinct clusters of CAGE peaks. Identifying the most frequently used dominant CTSSs allows for the positioning of the TSSs with single-base pair precision, which ideally corresponds to the gene annotation. To compare the position of these CTSSs with the MorexV3 annotated TSSs (aTSSs), we utilized dominant CTSSs from the primary set, ensuring that their distance from the aTSSs did not exceed 500 bp. This comparison revealed a notable degree of misalignment. Allowing a 20-bp deviation in either direction, 39% of expressed genes (detected by both CAGE and RNA-seq) concurred on the TSS position. Another 29% of aTSSs were located within 100 bp, while the remaining 31% were situated within 500 bp of the dominant CTSS (Fig. 1E). From this primary set, 2583 promoters were associated with the low-confidence gene category. Given these findings, the presence of a CAGE signal in close proximity to the aTSS could be a reason to reconsider the categorization for these specific genes. Supported by RNA-seq and epigenomic data, the CAGE signal enabled us to propose multiple putative unannotated genes, exemplified in Figure S1D.

A summary of tag counts within a CTSS serves as a measure of 5′ transcription initiation level, providing an index of gene expression quantified as tags per million. We conducted both qualitative (i.e., which genes were detected by either or both methods) and quantitative comparisons between CAGE and RNA-seq datasets. The quantitative analysis involved calculating correlations of tag/transcript counts for active genes detected by both methods (Figure S2A, B and Data S2). The comparison indicated that CAGE is limited in detecting low levels of gene expression (Figure S2C). Conversely, genes whose transcripts were detected by CAGE but not RNA-seq were not predominantly of low expression (Figure S2D), suggesting that discrepancies may arise from other biological or technical factors.

### 3.3 Promoter architecture clustering and the initiator

**Fig. 3:**
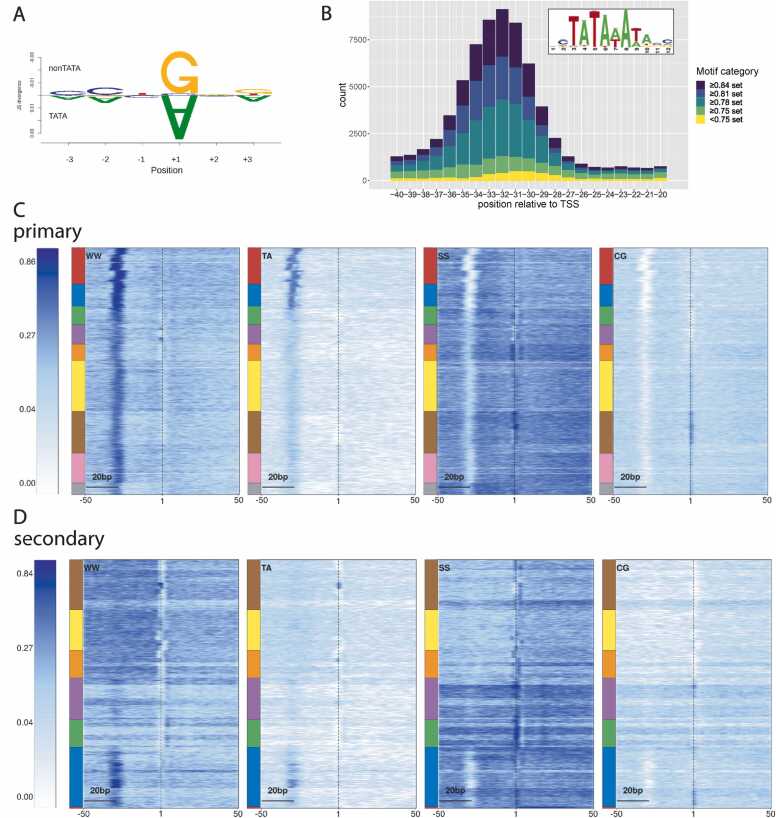
**Sequence analysis of the initiator, TATA-box and dinucleotide content in barley promoter clusters. A**) TATA vs. non-TATA box promoters differ in the + 1 position (A vs. G). **B**) Variability in the sequence and position of TATA-like motifs in the consensus promoter set. The motif categories are based on the degree of Pearson correlation with the canonical TATA-box position weight matrix (PWM). Pentamers included in the individual sets are listed in Table S3. **C**, **D**) Dinucleotide motif heat maps for the primary **C**) and the secondary **D**) promoter clusters. The heatmaps show enrichment of the given motif in the sequence with values 0–1. The multi-coloured bars left of the heat maps indicate boundaries between the clusters, ordered as in Figs. 2A and 2B, respectively. Note the presence of the W box in all primary promoters and the differences in CG distributions.

Based on previous extensive research on promoters of other species (reviewed in [45] and elsewhere), we posited that regions 50 bp up- and downstream of the dominant TSS likely contained a PIC-binding sequence. We employed seqArchR [46], a recently developed software that uses unsupervised approach using non-negative matrix factorization, to cluster promoter sequences based on their motifs at near-fixed distances from a reference point, such as TSS. These clusters are characterized by de novo-identified sequence elements, such as position-specific motifs and the nucleotide composition of the input sequences. Initially, we analyzed the primary TC sets from all three developmental stages, resulting in 15, 15, and 16 distinct sequence architecture clusters for 8DAP, 24DAP and 4DAG, respectively (Figure S3). In order to provide a generalized classification of promoter types across stages, we created a set of primary consensus promoters and subjected it to the seqArchR analysis. The resulting clusters, defined by their sequence architectures - including the initiator sequence, positioned TATA-box and other sequence motifs - and supplemented with the IQW and expression levels, were manually collated into fewer final clusters, ultimately settling to nine (Fig. 2A). The final number of clusters is the minimum number that still effectively differentiates the main TATA-box positions and other main architectures discovered in the default, non-collated serqArchR output. Further merging was tested but rejected because it would distort positionally restricted motifs, rendering them non-recognizable, and would merge distinct initiator configurations. The initiator, corresponding to the dominant CTSS and discernible as the sequence logo around positions − 1/+ 1, emerged as a highly significant motif in all clusters. Notably, barley lacks an extended initiator sequence motif, including the 'TCT' element, and this absence extends even to the promoters of ribosomal protein genes. The TATA-box containing promoters (clusters 1–3 of the primary set) are predominantly transcribed from a ‘CA’ initiator, which is associated mostly with genes exhibiting high expression levels. This underscores the pivotal role of the TATA box in dictating both the initiator sequence and promoter activity (i.e. transcriptional burst frequency). This feature is in stark contrast with the more varied pyrimidine-purine (PyPu) constellation ('CG' or 'TG'), prevalent in promoters lacking a distinct TATA box, which tend to exhibit lower expression levels (clusters 4–9, as shown in Figs. 2A and 3A).

The same clustering method was also applied to the secondary TCs (Fig. 2B). Seven distinct clusters best resolve all positioned motif configurations, which can be divided into three main groups. The TCs from clusters 1–3 originate from the first intron-exon boundary. These clusters are characterized by their pronounced sharpness and TPM values that are frequently higher than, and not synchronized with, the TPM of the primary TC of the hosting gene. These TCs are transcribed in the sense direction, and the base at the TSS corresponds mostly to one of the two bases forming the conserved eukaryotic splice acceptor site AG. An example is a gene coding for a 40 S ribosomal protein, which has been assigned to the secondary cluster 1 (Figure S4A). Conversely, secondary clusters 4 and 5 are characterized by both sense and antisense transcription initiated often at G-rich sequences originating at the exons, introns or intron-exon boundaries (Figure S4B). As there may be multiple secondary TCs per gene, gene ID redundancy and overlap between distinct clusters can occur. In the merged set of secondary clusters 1–3, out of a total of 6306 gene IDs, 4190 were unique. Similarly, in the merged set of secondary clusters 4–5, 2866 out of 3702 IDs were unique. Notably, there is an overlap of 1084 gene IDs between these two sets of clusters, which is statistically significant according to Fisher’s exact test (p-value of 0.000461). To determine whether this overlap might also indicate overlapping functions, we examined the GO enrichment in the 1084 genes with secondary TCs from both cluster sets 1–3 and 4–5. We isolated these genes from the rest of the unique gene IDs. The resulting Figure S8C demonstrates that the gene functions of these genes are confined to the same categories of metabolic and housekeeping functions that have been assigned to the original clusters. Interestingly, the genes with secondary TCs in both cluster sets 1–3 and 4–5 exhibit the highest enrichment levels.

Clusters 6 and 7 likely represent true alternative promoters, which are generally TATA-less, and promoters of potentially unannotated genes, as shown in Figure S1D.

### 3.4 The TATA box and other previously defined motifs in barley promoters

Consensus primary clusters 1–3, characterized by a positioned TATA-box-like motif, delineate the TSSs within significantly narrower regions (sharp promoters), in contrast to the broader regions associated with non-TATA promoters. This pattern is consistent across all studied organisms, although the position of the TATA box and the frequency of its occurrence do vary. To quantitatively analyze the TATA-box sequence with reproducibility, we utilized the position weight matrix (PWM) of the canonical plant TATA box (Fig. 3B inset). This PWM was generated from 134 unrelated plant promoter sequences deposited in the Eukaryotic Promoter Database [47]. A strand-specific search for the TATA-box frequency using this PWM and the FIMO tool [48] with the default p-value threshold of 1e-4 revealed that TATA boxes were present in 7.8%, 7.9%, and 9% of all promoters active at the 8DAP, 24DAP, and 4DAG stage, respectively. Employing a more lenient p-value of 1e-3, which included motifs diverging significantly from the canonical TATAWAW, the proportion of active promoters increased to 20%, 20%, and 23%, respectively.

**Fig. 4:**
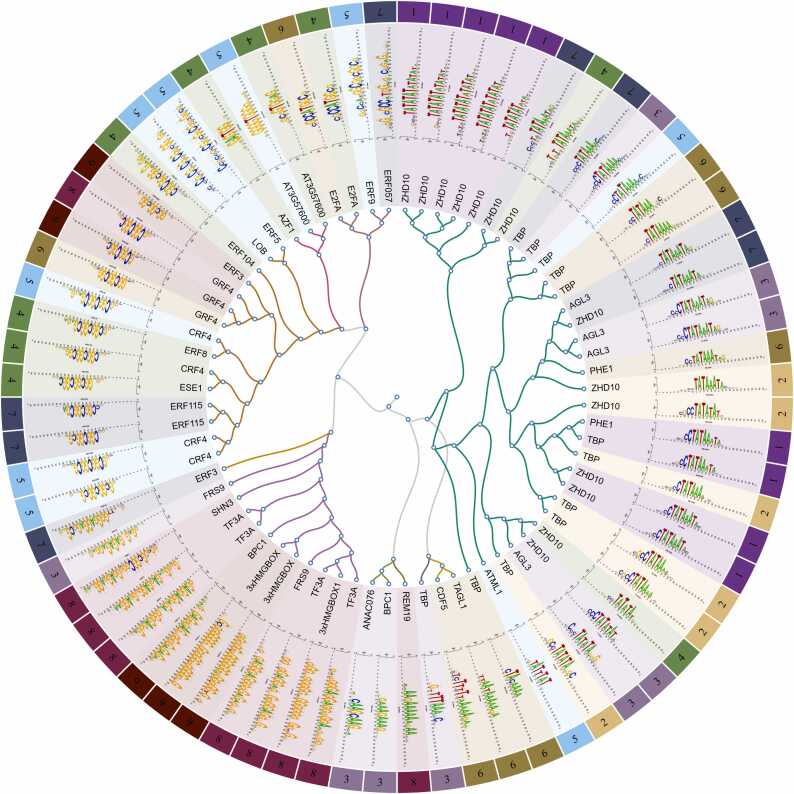
**Positioned sequence motifs in consensus core promoters.** Region + /− 50 bp relative to the TSS was searched for TFBS motifs collected in footprintDB.plants database. Proteins at the tree branches represent the motifs with the highest probability of occurrence identified in the search. The colors and numbers in the outer circle correspond to distinct primary promoter clusters.

Positional analysis using the same canonical PWM and TA pentamers as in [49] revealed that the starts of the TATA-box and more variable W-box motifs are located at − 29 to − 36 upstream of the initiator site, peaking at − 32. Greater distances were observed for the more conserved TATA-box motifs (Fig. 3B). A certain proportion of TATA-box promoters are associated with distinct cytosine nucleotides immediately upstream of the TATA box, as indicated by subsequent motif discovery (Fig. 4). This could suggest the presence of the BRE-upstream sequence (BREu, SSRCGCC), although neither the full BREu nor the BRE-downstream sequence (BREd, RTDKKKK) were detected (Figure S5). Apart from the TATA boxes and specific initiators, other previously recognized core promoter motifs were not observed (Figure S5). Nonetheless, dinucleotide heat maps produced by seqArchR provide a comprehensive view of the common PyPu dinucleotides at TSSs, which include the omnipresent W boxes (W=A/T) and a high content of SS dinucleotides (S=G/C) in the majority of barley promoters (Fig. 3C, D).

To detect less pronounced motifs within the core promoter regions of each cluster and to identify, which regulatory proteins might target them putatively, we supplemented the cluster analysis with an analysis using the RSAT toolkit [50]; https://github.com/rsa-tools/rsat-code). Specifically, we utilized a ‘peak-motif positioned’ function that detects oligonucleotides showing a positional bias, i.e. having a non-homogeneous distribution in the sequence set, followed by a TFBS search against footprintDB.plants [51]. For a comprehensive summary, the collection of motifs from all nine consensus clusters in the primary set underwent hierarchical clustering, which is depicted as a radial tree (Fig. 4, Data S5). This analysis confirmed that, in addition to the well-known canonical TATA box motif (TATAWAW), there is a significant occurrence (approximately 5.5% of cases, p-value = 1e-4) of extended TA repeats, which are sequences of 6–15 TA dinucleotides that do not align with the TBP matrix in footprintDB. Instead, these sequences seem to be targeted by the zinc finger protein ZHD10, which plays a role in establishing leaf polarity during development via gibberellic acid signaling pathway. Furthermore, other putative W-box motifs are consistent with the binding matrices of AGL3/PHE1, both of which are MADS-box homeotic transcription factors.

Additionally, other motifs within barley core promoters have a low-complexity nature, such as the previously described pyrimidine-rich Y patch [8], which has been experimentally demonstrated to enhance gene expression in the maize reporter system [29]. This motif, characterized by the sequence CTTCTTCCTC or its reverse complement GAGGAAGAAG, is present in over 14% of barley core promoters under strict search conditions (p-value ≤ 1e-5) and in up to 70% when using relaxed criteria (p-value ≤ 1e-4). The TFBS analysis conducted with RSAT attributes this motif to BPC1 (BASIC PENTACYSTEINE1), a GA-repeat-binding protein with an octodinucleotide binding preference, which has a homolog in barley known as BBR. This protein is thought to be involved in the Polycomb-mediated transcriptional regulation of developmental genes through its interaction with Polycomb Repressive Elements [52]. Other significant findings include 3xHMG-box proteins associated with cell proliferation and implicated in the organization of plant mitotic chromosomes [53], as well as hormone-responsive factors TF3A, FRS, and SHN3. Another group of low complexity motifs prevalent in barley core promoters is the GCC box, which is recognized by a GCC box-binding factor (GBF) and/or ethylene-responsive factors (ERFs); these are all hormone-responsive proteins implicated in stress responses and developmental processes. The analysis of an extended sequence range ( ± 100 bp) did not reveal any novel motifs (Figure S6). Other TF bindings in core promoters include E2F, MYB, and NAC transcription factors.

### 3.5 CT-rich motifs downstream TSSs might act as nutrient-sensing TOP motifs

**Fig. 5:**
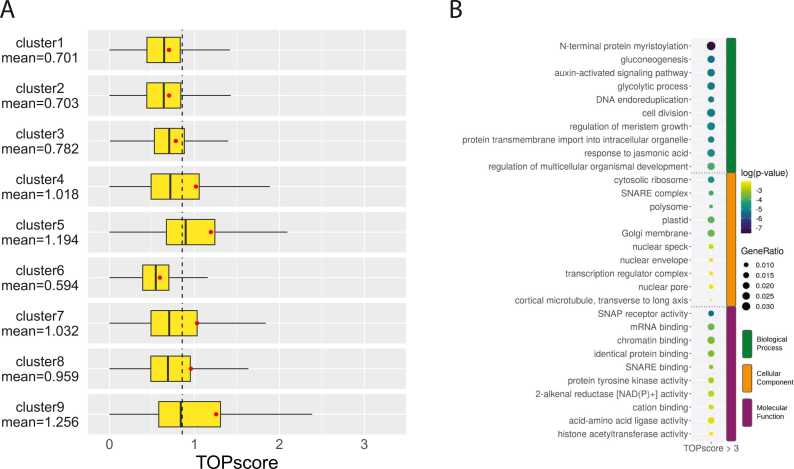
**Analysis of TOP Motifs Across Promoter Clusters. A**) Distributions of TOPscores in the primary consensus clusters. Each cluster exhibited either a significantly lower or higher TOPscore mean (represented by red dots) compared to the dataset's overall mean (mean=0.86, indicated by a dashed line), with statistical significance (p-value < 0.001, Welch Two-Sample t-test). **B**) GO enrichment analysis of genes with promoters containing the candidate TOP motif (TOPscore >3).

Although the search for TFBS within the promoter sequences yielded numerous matches, the region immediately downstream of the TSSs may function beyond transcriptional regulation. This region has been demonstrated to act as a translational signal, particularly via the TOP motif, which commences with a cytosine at the 5′ cap, followed by a sequence of uracils and/or cytosines, with few or no adenines or guanines. To investigate the potential of certain barley promoter sequences to serve as a platform for TOR signaling, we calculated TOPscore values for our CAGE dataset for 4DAG using the algorithm described in reference [35]. This revealed a set of mRNAs that begin with a likely 5′TOP motif as potential subjects to the TOR-5′TOP nutritional signaling pathway. Overall, the distribution and means of TOPscores for each cluster were significantly skewed (p < 0.001, Welch's two-sample t-test) toward lower values in TATA-box clusters 1–3 and cluster 6, while the remaining clusters displayed significantly higher values (Fig. 5A). A total of 558 barley genes had TOPscore values greater than 3, considered to carry the bona fide TOP motif signature in their 5′UTRs. GO term analysis of these candidate genes revealed significant enrichment in ribosomes, the Golgi apparatus, and plastids, with functional involvement primarily in sugar metabolism, cell division, and plant growth (Fig. 5B). This suggests that they are likely direct targets of the nutrient-sensing pathway.

### 3.6 Tissue specificity and GO enrichment analysis

To evaluate the relationship between gene features driven by specific promoters and the categories of these promoters, we initially examined the expression breadth or specificity of these genes throughout plant development and in various tissues. We computed a tau value for each gene using a TPM matrix encompassing a wide array of tissues as presented in the developmental transcriptomic dataset [40], which is accessible in the EoRNA database [54]. The distribution of tau values for individual promoter clusters again distinctly separates the clusters containing TATA boxes, which exhibit significantly higher tissue specificity, from those without TATA boxes, which are generally more ubiquitously expressed (Fig. 2A, Figure S7). Remarkably, the genes that display a pronounced capped-mRNA signal at the intron-exon junction, namely the TCs in secondary clusters 1–3, are the most ubiquitous (Fig. 2B).

To conduct a GO enrichment analysis, we generated a detailed plant-centered GO annotation using the GOMAP toolkit [36]. Compared to the published MorexV3 GO-term annotation [30], generated by the Automated Assignment of Human Readable Descriptions (AHRD) pipeline [55], GOMAP generated additional 3491 terms, assigning one to each barley gene, including those with a missing GO term in the published annotation (37% of the analyzed gene set). Annotation of several well-defined gene categories (e.g., histones, auxin-responsive genes, MADS-box) confirmed a better definition of gene functions compared to the published version (Figure S8A, B). The new barley GO-term annotation is available in GAF format as Data S3.

**Fig. 6:**
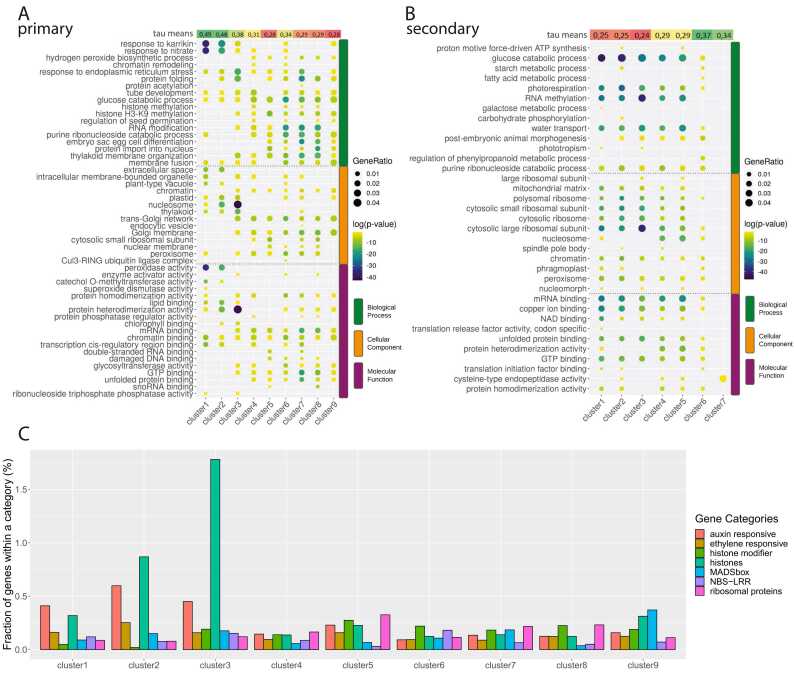
**GO enrichment analysis.** The plot shows the top five GO terms associated with primary **A**) and secondary **B**) consensus clusters. The uppermost row, labeled as 'tau means', indicates the tissue specificity scores for genes in each cluster. In panel A, Clusters 1–3 are identified as TATA-box clusters. **C**) illustrates the distribution of selected gene categories within the clusters of the primary set.

The enrichment analysis of GO terms within individual promoter clusters revealed that genes regulated by the most active and narrowly defined TATA-box promoters were annotated as responsive to environmental stimuli, stress, and signals related to hormonal, developmental, and organ growth processes (see Fig. 6A). These included a response to karrikin - a plant growth regulator structurally similar to strigolactones - implicated in seed germination, nitrate response, peroxidase activity, and protein folding. In a targeted approach, a manually curated subset of 116 genes responsive to auxin showed a clear association with the first three promoter clusters (Fig. 6C), as did genes encoding histones. Clusters 4–9, lacking a TATA box, exhibited a substantial overlap in functions attributable to basic cellular processes and some metabolic activities, as well as to translational regulation (including RNA modification and Golgi apparatus functions), exemplified by genes encoding ribosomal proteins (Fig. 6C). Notably, GO terms pertaining to transcriptional regulation - such as transcription factor binding to cis-regulatory regions or chromatin binding, which are typically linked with developmental genes - were represented in both categories of promoters in the primary dataset. Unexpectedly, secondary clusters 1–5 displayed a significant degree of functional similarity (Fig. 6B). These functions could be characterized as predominantly metabolic, related to ribosome structure and function, and involved in glucose metabolism, photorespiration, as well as RNA methylation and binding.

### 3.7 Promoter developmental shifts across barley embryo stages

**Fig. 7:**
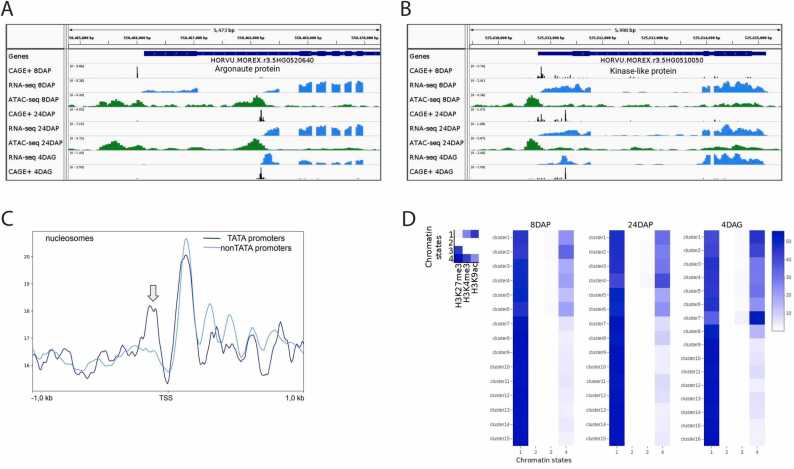
**Barley promoter shifts and epigenetic context. A**) A developmentally regulated alternative first exon in an Argonaute protein gene, accompanied by the dynamics of the epigenetic environment, as shown by open-chromatin profiles (green peaks) across three embryonic developmental stages. **B**) An example of an alternative TSS that shifts during development altering the 5`UTR sequence. The shift is validated by differences in RNA-seq and open-chromatin profiles. **C**) The nucleosome positioning for the TATA (merged clusters 1–3 of the primary consensus set) and non-TATA promoters (clusters 4–9). A well-positioned upstream nucleosome (indicated by an arrow) is correlated with the presence of the TATA box, contrasting with the nucleosome-free region and well-positioned downstream nucleosomes in TATA box-lacking promoters. **D**) Four chromatin states, inferred using ChromHMM from ChIP-seq datasets at 8DAP, 24DAP, and 4DAG, are based on three key histone modifications (left). Stage-specific heat maps (right) display the enrichment of these states across promoter clusters at different stages. The color key represents the fold enrichment of each chromatin state within each promoter cluster. Notably, the silencing H3K27me3 mark, predominantly found in chromatin states 3 and 4, shows higher enrichment in TATA-box clusters (i.e., clusters 1–6 for 8DAP and 24DAP, and clusters 1–7 for 4DAG), as further detailed in Fig. S3.

Altering promoter selection is one of the mechanisms for cell type differentiation. The use of an alternative promoter, which manifests as a distinct TC associated with the same gene, can coincide with a change in promoter architecture. This often involves switching between TATA-box and TATA-less promoter types. The putative alternative promoters identified in our study predominantly belong to secondary cluster 6. A subset of these alternative promoters is developmentally regulated, which we have termed "moving promoters”. The average length of promoter shift between stages examined in our study was approximately 500 bp, typically encompassing the 5′UTR, the first exon, or intron, thereby influencing the length of the UTR or the coding sequence. In our CAGEr consensus dataset, we sought instances of these shifts, considering only those TSS pairs that involved at least one of the following elements: promoter, 5′UTR, and promoter-proximal sequence (ranging from −500 to −1000 bp relative to the TSS). We then assessed the potential TSS shifts between embryonic stages based on the TPM values of each TC in the pair. Comparing pairs of stages with the highest stage-specific TPM values, we revealed 182, 154 and 160 genes with developmentally changed TSSs between the stage pairs 8DAPx4DAG, 8DAPx24DAP and 24DAPx4DAG, respectively (Data S4). Of these genes, 60, 34, and 51 involved the coding region, potentially altering the amino acid sequence, while 110, 115, and 103 affected the promoter/5′UTR region, potentially impacting transcriptional, translational, and transport signals. For instance, an alternative promoter in the first intron of an Argonaute protein gene is active in embryos at 4DAG and produces a transcript truncated at the 5′ end compared to the 8DAP stage. The transcription initiation alternatives were also reflected in the RNA-seq and Assay for Transposase-Accessible Chromatin using sequencing (ATAC-seq) data (Fig. 7A). Fig. 7B presents an example of a 5′UTR/promoter shift for a kinase-like protein gene.

### 3.8 Epigenetic characteristics of barley promoters

The two primary modes of transcription regulation - TATA-dependent, characterized by sharp promoters (or TCs), and TATA-independent, associated with broad promoters (or TCs) - are known to correlate with distinct promoter-proximal nucleosome positioning in metazoans [56]. To investigate the relationship between nucleosome positioning and barley promoter architectures, we utilized data from MNase-digested DNA sequencing of 24DAP embryos. Resulting plots of nucleosome distributions show a dominant nucleosome immediately downstream of the TSS - a characteristic shared by all promoters - but also two distinct profiles for other adjacent nucleosomes (Fig. 7C, detailed in Figure S9). In the region upstream of the TSS, the narrower TATA-box promoters exhibit a well-positioned nucleosome, as opposed to non-TATA promoters, which display a nucleosome-free region. Conversely, several nucleosomes downstream of the TSS appear to be very precisely positioned in the latter category of promoters but are less well-positioned in the TATA-box group.

The key promoter nucleosomes are typically marked by histone-3 post-translational modifications, specifically H3K4me3 and H3K9ac, in active genes. We conducted native chromatin immunoprecipitation (ChIP) using antibodies specific to histone modifications, including those two activating ones as well as the Polycomb- and facultative heterochromatin-related H3K27me3. K-means clustering of each cluster profile revealed that TATA clusters contained two distinct composite profiles: H3K4me3 plus H3K9ac and H3K4me3 plus H3K27me3, resembling the bivalent marks considered to poise the expression of developmental genes (Figure S10A, B). In contrast, the silencing H3K27me3 mark was almost absent from the non-TATA promoters, which aligns with their lower tissue specificity. Chromatin-state (CS) analysis using ChromHMM [57] integrates genome-wide profiles of multiple histone modifications obtained by ChIP-seq. This method employs combinatorial and spatial patterns of marks to annotate each tissue sample. We utilized ChromHMM to model the presence or absence of each of the three histone modifications, thereby learning the CSs. These states were then applied to generate annotations for each tissue/stage, determining the most probable CS for each genomic segment. Finally, we performed enrichment analysis on these annotations for individual stage-specific clusters (Fig. 7D). This analysis confirmed that all TATA-box promoter clusters contained both activating marks (H3K4me3 and H3K9ac), as well as, albeit to a lesser extent, the silencing H3K27me3 mark. Notably, the proportion of promoters bearing the silencing mark was observed to increase concomitantly with cell differentiation.

An important feature of promoters, which is closely related to the epigenetic landscape, is their transposable element (TE) content. In the human genome, approximately 18% of start sites have been defined by CAGE as overlapping with TEs [58]. Intriguingly, an inspection of the degree of overlap between TEs, as annotated in [40], [59], and regions within + /− 50 bp of TSSs revealed that only 5–6% of TATA box-containing promoters overlapped with TEs. In contrast, 10% (cluster 6) to 15% (cluster 5) TE-overlapping promoters in the non-TATA category did overlap, as shown in Figure S10C). Predominant TE families included DNA transposons *CACTA* and retrotransposons such as *Copia* and *Gypsy*, along with others in the LTR-RLX class that are unknown. The overlap of these elements with promoters raises new questions about their role in plant gene regulation, development, and evolution.

## 4 Discussion

We investigated barley core promoter sequences utilizing precisely delineated transcription start sites identified by CAGE and employing a novel, unbiased data analysis approach. This approach accounts for the positional restrictions of motifs within the promoters and categorizes them based on their overall sequence architecture. It is independent of pre-existing motif knowledge and circumvents the detection of non-conserved noise, which typically arises from the examination of inaccurate promoter sequences and non-positioned motifs. Moreover, our method does not rely on the previously emphasized distinction between sharp and broad promoters, eliminating the need to define a boundary between the two categories. Still, our findings align with those from studies on metazoan promoteromes [5], indicating that the presence of the TATA box is crucial for governing TSS selection within a more constrained region associated with the CA initiator. Conversely, the presence of other W-box sequences, or their absence, appears to result in a more relaxed or flexible TSS selection that includes various PyPu initiator configurations.

Although the applied methods vary slightly, the proportion of promoters containing a relaxed form of the TATA-box-like motif was found to be approximately 20% in both the barley and the human promoterome [60]. A notably higher proportion (38%) was observed in maize genes active in both root and shoot tissues [22], which may reflect the fact that the TATA-box promoters are associated with tissue-specific expression primarily in adult tissues [61], as opposed to the early developmental stages analyzed in our study.

The TATA-box position is not strictly conserved across species, and its distance from the initiator element does not appear to be directly proportional to genome size. In barley, the TATA box is found within a wider range (from −29 to −36 nucleotides upstream of the TSS), which is slightly shifted in comparison to the positions commonly observed in metazoan genomes. In metazoans, the TATA box is typically located at a more constrained position, usually at the 30th or 31st nucleotide upstream of the TSS [60]. This variation may be associated with wider transcriptional core sequences (TCs) linked to the TATA box in plant species as opposed to those in animals. Similar spacing has been reported in Arabidopsis [29], approximately at − 30 nucleotides upstream of the TSS, and in maize, sorghum, rice, and wheat at − 34 nucleotides [9], indicating some degree of conservation among plant species.

In our observations, we identified a W-box variant that does not conform to the consensus sequence of the TBP-motif position weight matrix. This raises the question of whether factors other than TBP, evolutionarily related or not, can substitute for this essential protein in the pre-initiation complex, as predicted by motif analysis tools. Conversely, it is also worth investigating whether TBP can bind to motifs diverging from the TATA box, as has been reported in yeast [62]. In addition, the BREu, a C-rich element located upstream of the TATA box, was found to enhance promoter activity in maize when inserted into an artificial promoter in a transgenic assay. This effect contrasts with findings in tobacco and is particularly notable given that the motif is reportedly absent in maize [29]. We found only a partial match to this element (the ‘CC’ sequence), leaving its significance in barley to be functionally tested.

The two principal TSS selection modes are associated with nucleosome positioning, tissue specificity, and the epigenetic profile. These align with the concept of two distinct regulatory environments. However, the intricate interplay between plant hormones—which perform both developmental and housekeeping functions—and the inherent plasticity of plant cells, obscures the distinction between housekeeping and developmental gene promoters. This is probably reflected in our GO term analysis. In relation to this, the bivalent chromatin states identified in TATA-box-containing promoters facilitate timely activation while also enabling repression in the absence of differentiation signals [63]. Given the relatively complex nature of our samples, the presence of H3K27me3 could also be indicative of the repression of tissue-specific promoters in a subset of cell types. Therefore, the bivalent histone modification status should be confirmed or ruled out through a sequential ChIP experiment.

Our comparison of the dominant TSSs from CAGE datasets to the most recent (MorexV3) barley annotation resulted in relatively frequent misalignment: 61% CTSSs were located over 20 bp and 32% more than 100 bp distant from the aTSS. This discrepancy may be partly attributed to promoter shifts between tissues and developmental stages, given that the embryonal samples analyzed in our study were only marginally represented in the RNA-seq dataset [40] utilized for the MorexV3 annotation. This underscores the importance of creating a comprehensive promoterome atlas across multiple tissues and cell types for a given organism. Such an atlas would make it possible to focus on the relevant regulatory regions in gene cloning and editing projects. In this context, CAGE stands out as a cost-effective complementary technology capable of significantly enhancing even high-quality genome annotations that are primarily based on RNA-seq data.

To understand the differences between CAGE and RNA-seq, it is essential to recognize that these methodologies target disparate segments of RNA molecules: specifically, the capped 5′ ends and random RNA fragments, respectively [64]. The decreased sensitivity of CAGE is likely due to the TPM threshold applied to filter out widespread, yet low-level, intergenic signals, regardless of whether they are background noise or actual transcriptional events. Additionally, the depth of sequencing may have also contributed to this limitation. The requisite sequencing depth for barley was extrapolated from numerous studies conducted on human cells, which may have resulted in an underestimation. Such an underestimation can occur due to the broader promoter regions, along with distinct levels of intergenic transcription and background noise present in barley compared to those in human cells.

In comparison to other cereals, barley exhibited a smaller proportion, approximately 60,5%, of transcription start regions (TSRs) located in gene-proximal regions, while rice, maize, and sorghum had approximately 69–74% and wheat 49–54% of TSRs within a 1,000-bp distance from annotated genes [9]. These differences may be attributed to variations in detection techniques (CAGE versus Smar2C2) and data processing pipelines or could potentially signify differences in numbers of unannotated genes in the cereal annotation.

In addition to transcripts that overlap with annotated promoters, we identified over 2000 predominantly single-base-wide CTSSs emanating from the first intron-exon junction at the splice acceptor site. Similar transcripts, transcribed in the same direction as the gene, have been previously observed in mammalian CAGE datasets and are characterized as products of post-transcriptional cleavage and recapping. It has been hypothesized that these transcripts give rise to truncated mRNA isoforms that could potentially be translated into proteins with truncated C- or N-termini. [65]. The authors referred to these as 'intraexonic CAGE tags' and observed that they tend to manifest as tissue-specific variants, indicating a highly regulated process that presumably augments mRNA abundance. Alternatively, these CTSSs may arise as artifacts of co-transcriptional splicing or due to a deceleration and subsequent re-capping of RNA polymerase or may be a subject to post-transcriptional cleavage by Argonaute and used as regulatory non-coding RNA [66]. Lastly, they might result from intron-dependent loop formation, which is associated with a mechanism of transcriptional enhancement, as reviewed in [65]. Further characterization of these transcripts through rapid amplification of cDNA ends (RACE), RNA-binding protein immunoprecipitation, or the detection of corresponding translation products at the protein level would be a valuable extension of this research.

Our results demonstrate that in barley, the core promoter motifs identifiable are limited to the PyPu initiator, the W box, and the Y patch - previously established elements - supplemented by stretches of low-complexity sequences that exhibit some dynamic behavior and flexibility during development. The promoter sequence architecture seems to evolve through the exaptation of novel regions, including transposable elements, or via alterations in ancient promoters due to degeneration and changes in binding preferences. We posit that ancient cellular functions are more commonly orchestrated by sequences containing the TATA box and simple TF-binding motifs, whereas more specialized functions tend to be associated with less conserved promoter sequences. These less conserved sequences may facilitate polymerase scanning [67], interaction with co-activators, and initiation in response to distal TFBS.

Artificial promoter design and regulatory sequence manipulation are common engineering methods to drive or influence transcription levels. The knowledge of PIC interactions have been exploited by humans to create a highly active core promoter, termed the ‘super core promoter’, that is capable of engaging in high-affinity interactions with TFIID through the presence of optimal versions of the TATA, Inr, MTE, and DPE motifs [17]. Similarly, a plant ‘super-promoter’ has been designed by [29] who also indicated that for optimal results, species-specific promoters might be preferable in transgenic designs. Although functional validation of each individual promoter type is beyond the scope of this article, such validation can be readily addressed in the future using in vitro assays with a reporter gene, including tests for hormone, stress, and nutrient effects on promoter activity.

Our study, conducted on developing and germinating barley embryos, yielded comprehensive information regarding the initiation of transcription in 21,610 genes active during these specific stages. To enhance this dataset and obtain a more complete understanding of transcriptional regulation in barley, we plan to generate and analyze additional CAGE datasets, particularly from floral tissues, which are expected to reveal generative-tissue-specific regulatory mechanisms. Furthermore, we hypothesize that species-specific promoter models, constructed from a limited number of tissue-specific CAGE datasets, could facilitate genome-wide promoter prediction without necessitating the production of an exhaustive array of new datasets [68].

## Declaration of generative AI in scientific writing

During the preparation of this work the author(s) used chatGPT in order to correct English grammar and refine scientific style without changing text content. After using this tool/service, the author(s) reviewed and edited the content as needed and take(s) full responsibility for the content of the publication.

## Funding

This project has been supported by the 10.13039/501100001824Czech Science Foundation [grant number 21–18794S] and by the 10.13039/501100008530European Regional Development Fund project “Plants as a tool for sustainable global development” [No. CZ.02.1.01/0.0/0.0/16_019/0000827].

Computational resources were provided by the e-INFRA CZ project (ID:90140), supported by the Ministry of Education, Youth and Sports of the Czech Republic.

## CRediT authorship contribution statement

P.N. and H.S.: Project conceptualization; P.N., S.P.: Investigation; S.N., B.L. and S.P: Software design; S.P., P.N., and T.A. Formal analysis; M.K.: Resources (RNA-seq data); P.N., S.P.: Writing - Original Draft; P.N., H.S. and S.P.: Writing - Review & Editing. H.S.: Funding acquisition.

## Declaration of Competing Interest

The authors declare that they have no known competing financial interests or personal relationships that could have appeared to influence the work reported in this paper.

## Appendix A Supplementary material

.

**Data S1:** A complete list of the TCs in the sense directions according to the annotated genes, including consensus-TC coordinates in the MorexV3 genome, the assigned-gene ID, position of the dominant TSS, feature annotation, classification, IQW and TPM. **Data S2:** Interactive correlation plots of CAGE and RNA-seq. **Data S3:** The new barley GO-term annotation in GAF format. **Data S4:** Genes with developmental TSS shifts between embryonic stages. **Data S5:** Interactive result from hierarchical matrix clustering of motifs found in all barley promoters.

## References

[1] Burley S.K., Roeder R.G. (1996). Biochemistry and structural biology of transcription factor IID (TFIID). Annu Rev Biochem.

[2] Bergman D.T., Jones T.R., Liu V., Ray J., Jagoda E., Siraj L. (2022). Compatibility rules of human enhancer and promoter sequences. Nature.

[3] Martinez-Ara M., Comoglio F., van Arensbergen J., van Steensel B. (2022). Systematic analysis of intrinsic enhancer-promoter compatibility in the mouse genome. Mol Cell.

[4] Neumayr C., Haberle V., Serebreni L., Karner K., Hendy O., Boija A. (2022). Differential cofactor dependencies define distinct types of human enhancers. Nature.

[5] Haberle V., Lenhard B. (2016). Promoter architectures and developmental gene regulation. Semin Cell Dev Biol.

[6] Vo Ngoc L., Wang Y.-L., Kassavetis G.A., Kadonaga J.T. (2017). The punctilious RNA polymerase II core promoter. Genes Dev.

[7] Smale S.T., Baltimore D. (1989). The “initiator” as a transcription control element. Cell.

[8] Yamamoto Y.Y., Ichida H., Matsui M., Obokata J., Sakurai T., Satou M. (2007). Identification of plant promoter constituents by analysis of local distribution of short sequences. BMC Genom.

[9] Murray A., Mendieta J.P., Vollmers C., Schmitz R.J. (2022). Simple and accurate transcriptional start site identification using Smar2C2 and examination of conserved promoter features. Plant J.

[10] Bernard V., Brunaud V., Lecharny A. (2010). TC-motifs at the TATA-box expected position in plant genes: a novel class of motifs involved in the transcription regulation. BMC Genom.

[11] Cordon-Obras C., Gomez-Liñan C., Torres-Rusillo S., Vidal-Cobo I., Lopez-Farfan D., Barroso-Del Jesus A. (2022). Identification of sequence-specific promoters driving polycistronic transcription initiation by RNA polymerase II in trypanosomes. Cell Rep.

[12] Marbach-Bar N., Bahat A., Ashkenazi S., Golan-Mashiach M., Haimov O., Wu S.-Y. (2016). DTIE, a novel core promoter element that directs start site selection in TATA-less genes. Nucleic Acids Res.

[13] Danks G.B., Navratilova P., Lenhard B., Thompson E.M. (2018). Distinct core promoter codes drive transcription initiation at key developmental transitions in a marine chordate. BMC Genom.

[14] Shao W., Alcantara S.G.-M., Zeitlinger J. (2019). Reporter-ChIP-nexus reveals strong contribution of the Drosophila initiator sequence to RNA polymerase pausing. eLife.

[15] Vo Ngoc L., Cassidy C.J., Huang C.Y., Duttke S.H.C., Kadonaga J.T. (2017). The human initiator is a distinct and abundant element that is precisely positioned in focused core promoters. Genes Dev.

[16] Menand B., Desnos T., Nussaume L., Berger F., Bouchez D., Meyer C. (2002). Expression and disruption of the Arabidopsis TOR (target of rapamycin) gene. Proc Natl Acad Sci USA.

[17] Cianfrocco M.A., Kassavetis G.A., Grob P., Fang J., Juven-Gershon T., Kadonaga J.T. (2013). Human TFIID Binds to Core Promoter DNA in a Reorganized Structural State. Cell.

[18] Carninci P., Sandelin A., Lenhard B., Katayama S., Shimokawa K., Ponjavic J. (2006). Genome-wide analysis of mammalian promoter architecture and evolution. Nat Genet.

[19] Deal R.B., Henikoff S. (2011). Histone variants and modifications in plant gene regulation. Curr Opin Plant Biol.

[20] Vermeulen M., Mulder K.W., Denissov S., Pijnappel W.W.M.P., van Schaik F.M.A., Varier R.A. (2007). Selective anchoring of TFIID to nucleosomes by trimethylation of histone H3 lysine 4. Cell.

[21] Haberle V., Li N., Hadzhiev Y., Plessy C., Previti C., Nepal C. (2014). Two independent transcription initiation codes overlap on vertebrate core promoters. Nature.

[22] Mejía-Guerra M.K., Li W., Galeano N.F., Vidal M., Gray J., Doseff A.I. (2015). Core Promoter Plasticity Between Maize Tissues and Genotypes Contrasts with Predominance of Sharp Transcription Initiation Sites. Plant Cell.

[23] Shiraki T., Kondo S., Katayama S., Waki K., Kasukawa T., Kawaji H. (2003). Cap analysis gene expression for high-throughput analysis of transcriptional starting point and identification of promoter usage. Proc Natl Acad Sci USA.

[24] Yamamoto Y.Y., Yoshitsugu T., Sakurai T., Seki M., Shinozaki K., Obokata J. (2009). Heterogeneity of Arabidopsis core promoters revealed by high-density TSS analysis. Plant J.

[25] Core L.J., Waterfall J.J., Lis J.T. (2008). Nascent RNA sequencing reveals widespread pausing and divergent initiation at human promoters. Science.

[26] Carninci P., Kasukawa T., Katayama S., Gough J., Frith M.C., Maeda N. (2005). The transcriptional landscape of the mammalian genome. Science.

[27] FANTOM Consortium and the RIKEN PMI and CLST (DGT), Forrest A.R.R., Kawaji H., Rehli M., Baillie J.K., de Hoon M.J.L. (2014). A promoter-level mammalian expression atlas. Nature.

[28] Thieffry A., Vigh M.L., Bornholdt J., Ivanov M., Brodersen P., Sandelin A. (2020). Characterization of Promoter Bidirectionality and Antisense RNAs by Inactivation of Nuclear RNA Decay Pathways. Plant Cell.

[29] Jores T., Tonnies J., Wrightsman T., Buckler E.S., Cuperus J.T., Fields S. (2021). Synthetic promoter designs enabled by a comprehensive analysis of plant core promoters. Nat Plants.

[30] Mascher M., Wicker T., Jenkins J., Plott C., Lux T., Koh C.S. (2021). Long-read sequence assembly: a technical evaluation in barley. Plant Cell.

[31] Navrátilová P., Toegelová H., Tulpová Z., Kuo Y.-T., Stein N., Doležel J. (2022). Prospects of telomere-to-telomere assembly in barley: Analysis of sequence gaps in the MorexV3 reference genome. Plant Biotechnol J.

[32] Schreiber M., Mascher M., Wright J., Padmarasu S., Himmelbach A., Heavens D. (2020). A Genome Assembly of the Barley “Transformation Reference” Cultivar Golden Promise. G3.

[33] Kovacik M., Nowicka A., Pecinka A. (2020). Isolation of High Purity Tissues from Developing Barley Seeds. J Vis Exp.

[34] Yu G., Wang L.-G., He Q.-Y. (2015). ChIPseeker: an R/Bioconductor package for ChIP peak annotation, comparison and visualization. Bioinformatics.

[35] Philippe L., van den Elzen A.M.G., Watson M.J., Thoreen C.C. (2020). Global analysis of LARP1 translation targets reveals tunable and dynamic features of 5′ TOP motifs. Proc Natl Acad Sci.

[36] Wimalanathan K., Lawrence-Dill C.J. Gene Ontology Meta Annotator for Plants (GOMAP). doi:10.1101/809988. 10.1186/s13007-021-00754-1 PMC8146647 34034755

[37] Supek F., Bošnjak M., Škunca N., Šmuc T. (2011). REVIGO summarizes and visualizes long lists of gene ontology terms. PLoS One.

[38] Neumann P., Navrátilová A., Schroeder-Reiter E., Koblížková A., Steinbauerová V., Chocholová E. (2012). Stretching the rules: monocentric chromosomes with multiple centromere domains. PLoS Genet.

[39] Ernst J., Kellis M. (2017). Chromatin-state discovery and genome annotation with ChromHMM. Nat Protoc.

[40] Mascher M., Gundlach H., Himmelbach A., Beier S., Twardziok S.O., Wicker T. (2017). A chromosome conformation capture ordered sequence of the barley genome. Nature.

[41] Kovacik M., Nowicka A., Zwyrtková J., Strejčková B., Vardanega I., Esteban E. (2023). The transcriptome landscape of developing barley seeds reveals H3K27me3 dynamics in endosperm tissues. bioRxiv.

[42] Applied Research Applied Research Press. RSEM: Accurate Transcript Quantification from RNA-Seq Data with Or Without a Reference Genome. 2015. 10.1186/1471-2105-12-323 PMC3163565 21816040

[43] Li B., Dewey C.N. (2011). RSEM: accurate transcript quantification from RNA-Seq data with or without a reference genome. BMC Bioinforma.

[44] Haberle V., Forrest A.R.R., Hayashizaki Y., Carninci P., Lenhard B. (2015). CAGEr: precise TSS data retrieval and high-resolution promoterome mining for integrative analyses. Nucleic Acids Res.

[45] Levine M., Tjian R. (2003). Transcription regulation and animal diversity. Nature.

[46] Nikumbh S., Lenhard B. (2023). Identifying promoter sequence architectures via a chunking-based algorithm using non-negative matrix factorisation. PLoSComputBiol.

[47] Cavin Périer R., Junier T., Bucher P. (1998). The Eukaryotic Promoter Database EPD. Nucleic Acids Res.

[48] Grant C.E., Bailey T.L., Noble W.S. (2011). FIMO: scanning for occurrences of a given motif. Bioinformatics.

[49] Wragg J.W., Roos L., Vucenovic D., Cvetesic N., Lenhard B., Müller F. (2020). Embryonic tissue differentiation is characterized by transitions in cell cycle dynamic-associated core promoter regulation. Nucleic Acids Res.

[50] Santana-Garcia W., Castro-Mondragon J.A., Padilla-Gálvez M., Nguyen N.T.T., Elizondo-Salas A., Ksouri N. (2022). RSAT 2022: regulatory sequence analysis tools. Nucleic Acids Res.

[51] Sebastian A., Contreras-Moreira B. (2014). footprintDB: a database of transcription factors with annotated cis elements and binding interfaces. Bioinformatics.

[52] Xiao J., Jin R., Yu X., Shen M., Wagner J.D., Pai A. (2017). Cis and trans determinants of epigenetic silencing by Polycomb repressive complex 2 in Arabidopsis. Nat Genet.

[53] Pedersen D.S., Coppens F., Ma L., Antosch M., Marktl B., Merkle T. (2011). The plant-specific family of DNA-binding proteins containing three HMG-box domains interacts with mitotic and meiotic chromosomes. N Phytol.

[54] Milne L., Bayer M., Rapazote-Flores P., Mayer C.-D., Waugh R., Simpson C.G. (2021). EORNA, a barley gene and transcript abundance database. Sci Data.

[55] Boecker F. AHRD: Automatically annotate proteins with human readable descriptions and Gene Ontology terms. Universitäts- und Landesbibliothek Bonn. 2021. Available: https://bonndoc.ulb.uni-bonn.de/xmlui/handle/20.500.11811/9344.

[56] Rach E.A., Winter D.R., Benjamin A.M., Corcoran D.L., Ni T., Zhu J. (2011). Transcription initiation patterns indicate divergent strategies for gene regulation at the chromatin level. PLoS Genet.

[57] Ernst J., Kellis M. (2012). ChromHMM: automating chromatin-state discovery and characterization. Nat Methods.

[58] Djebali S., Davis C.A., Merkel A., Dobin A., Lassmann T., Mortazavi A. (2012). Landscape of transcription in human cells. Nature.

[59] Wicker T., Schulman A.H., Tanskanen J., Spannagl M., Twardziok S., Mascher M. (2017). The repetitive landscape of the 5100 Mbp barley genome. Mob DNA.

[60] Ponjavic J., Lenhard B., Kai C., Kawai J., Carninci P., Hayashizaki Y. (2006). Transcriptional and structural impact of TATA-initiation site spacing in mammalian core promoters. Genome Biol.

[61] Haberle V., Stark A. (2018). Eukaryotic core promoters and the functional basis of transcription initiation. Nat Rev Mol Cell Biol.

[62] Seizl M., Hartmann H., Hoeg F., Kurth F., Martin D.E., Söding J. (2011). A Conserved GA Element in TATA-Less RNA Polymerase II Promoters. PLoS One.

[63] Voigt P., Tee W.-W., Reinberg D. (2013). A double take on bivalent promoters. Genes Dev.

[64] Kawaji H., Lizio M., Itoh M., Kanamori-Katayama M., Kaiho A., Nishiyori-Sueki H. (2014). Comparison of CAGE and RNA-seq transcriptome profiling using clonally amplified and single-molecule next-generation sequencing. Genome Res.

[65] Mercer T.R., Dinger M.E., Bracken C.P., Kolle G., Szubert J.M., Korbie D.J. (2010). Regulated post-transcriptional RNA cleavage diversifies the eukaryotic transcriptome. Genome Res.

[66] Haberman N., Digby H., Faraway R., Cheung R., Jobbins A.M., Parr C. (2023). Abundant capped RNAs are derived from mRNA cleavage at 3′UTR G-Quadruplexes. bioRxiv.

[67] Qiu C., Jin H., Vvedenskaya I., Llenas J.A., Zhao T., Malik I. (2020). Universal promoter scanning by Pol II during transcription initiation in Saccharomyces cerevisiae. Genome Biol.

[68] Wang Y., Peng Q., Mou X., Wang X., Li H., Han T. (2022). A successful hybrid deep learning model aiming at promoter identification. BMC Bioinforma.

