# Supplementary material for: Core promoterome of barley embryo
Source: Comput Struct Biotechnol J. 2023 Dec 5;23:264–77. doi: 10.1016/j.csbj.2023.12.003 (PMC10762323; doi:10.1016/j.csbj.2023.12.003)
Supplement: Supplementary file 1 — Supplementary material [file mmc1.docx]

**Supplemental Material**

**F**

**igure S1. CAGE data initial analysis: replica correlation, CAGEr analysis settings and TC annotation across stages.**

**A**) Correlation of mapped barley CAGE data (BAM files) that were used for CAGEr analysis. **B**) Reverse cumulative distribution plot with fitted power-law distributions showing the range on which the CAGEr power-law normalization settings were based (alpha = 1.05, T = 1e+06, TPM threshold = 0.1, fitInRange = c(5, 10000)) **C**) Location of CTSS clusters across genome features as annotated using ChIPseeker`s annotatePeak function. The plot shows the annotations for three embryo stages (8DAP/24DAP/4DAG) before they were splitted into primary and secondary promoter datasets. **D**) An example of a putative unannotated gene detected by CAGE and RNA-seq in all three embryo stages. ATAC-seq peaks and chromatin state characterized by the presence of activating histone modifications (red bar) evidence the transcriptional activity of the sequence.


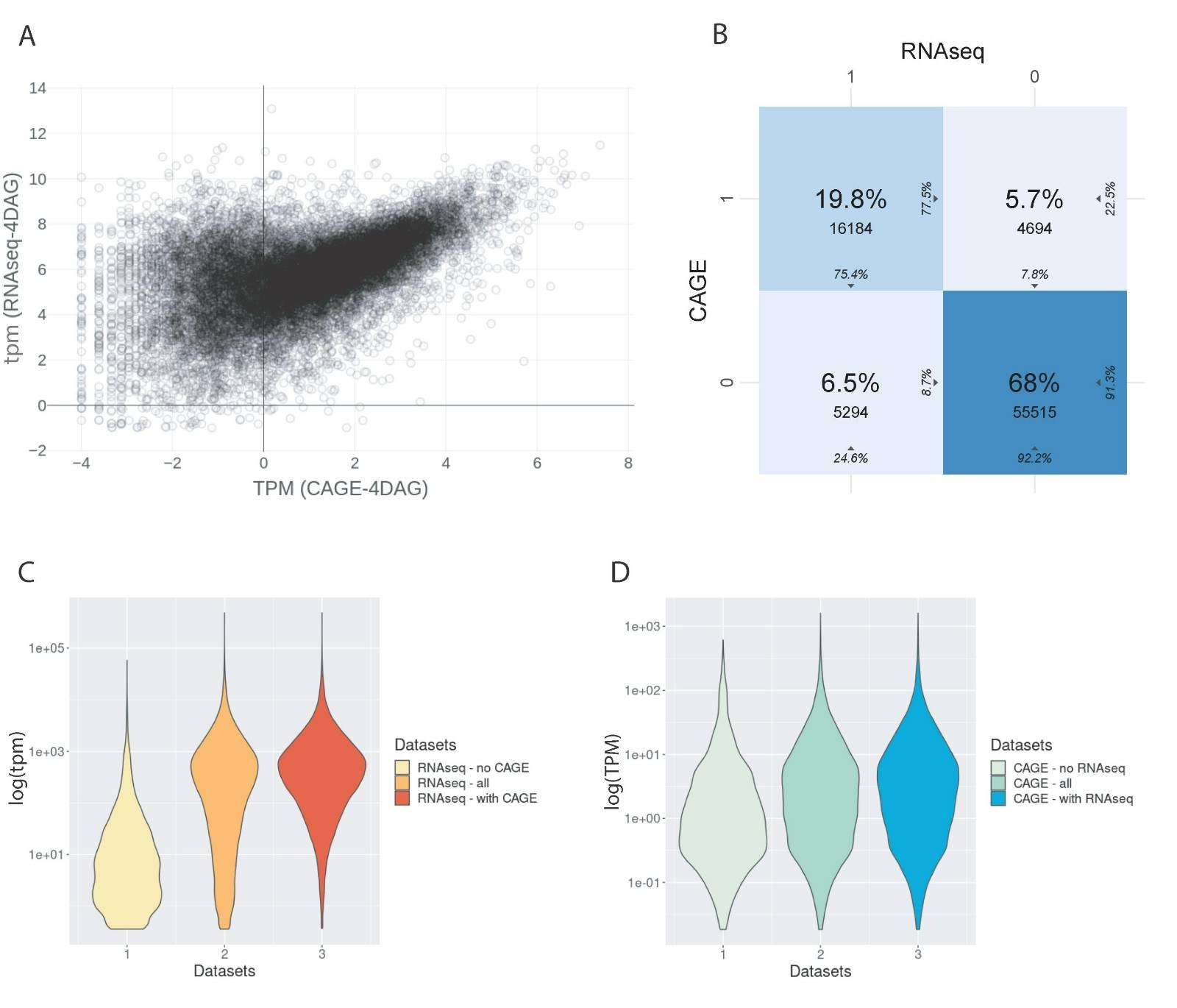


**Figure S2. Comparison of 4DAG CAGE and RNAseq datasets.** **A**) Correlations of 4DAG RNAseq/CAGE data based on the decimal logarithm of TPM; B) CAGE/RNAseq confusion matrix showing overlap of the expressed gene sets detected by the two methods against the whole set of 81,683 annotated genes in the MorexV3 assembly. **C**) RNAseq tpm comparison between datasets of the 4DAG sample. Dataset 1 = tpm of RNAseq-predicted promoters that were not identified by CAGE, Dataset 2 = mean of tpm for complete RNAseq dataset, Dataset 3 = TPM of all RNAseq-predicted promoters that were also identified by CAGE. **D**) CAGE TPM comparison between datasets of the 4DAG sample. Dataset 1 = TPM of CAGE-predicted promoters that were not identified by RNAseq, Dataset 2 = mean of TPM for complete CAGE dataset, Dataset 3 = TPM of all CAGE-predicted promoters that were also identified by RNAseq. The RNAseq data were obtained from [40].





**Figure S3. Clustering of 8DAP, 24DAP and 4DAG CAGE promoter sequence architectures.** Clusters of stage-specific primary promoters were generated by the seqArchR algorithm and ordered by median interquantile widths (IQWs). The composed plots for **A**) 8DAP, **B**) 24DAP and **C**) 4DAG samples include boxplots for IQW and gene expression level values (tags per million (TPM), log-transformed), followed by sequence logos and genomic feature annotation bar plots. The numbers of genes per cluster are given in parentheses.


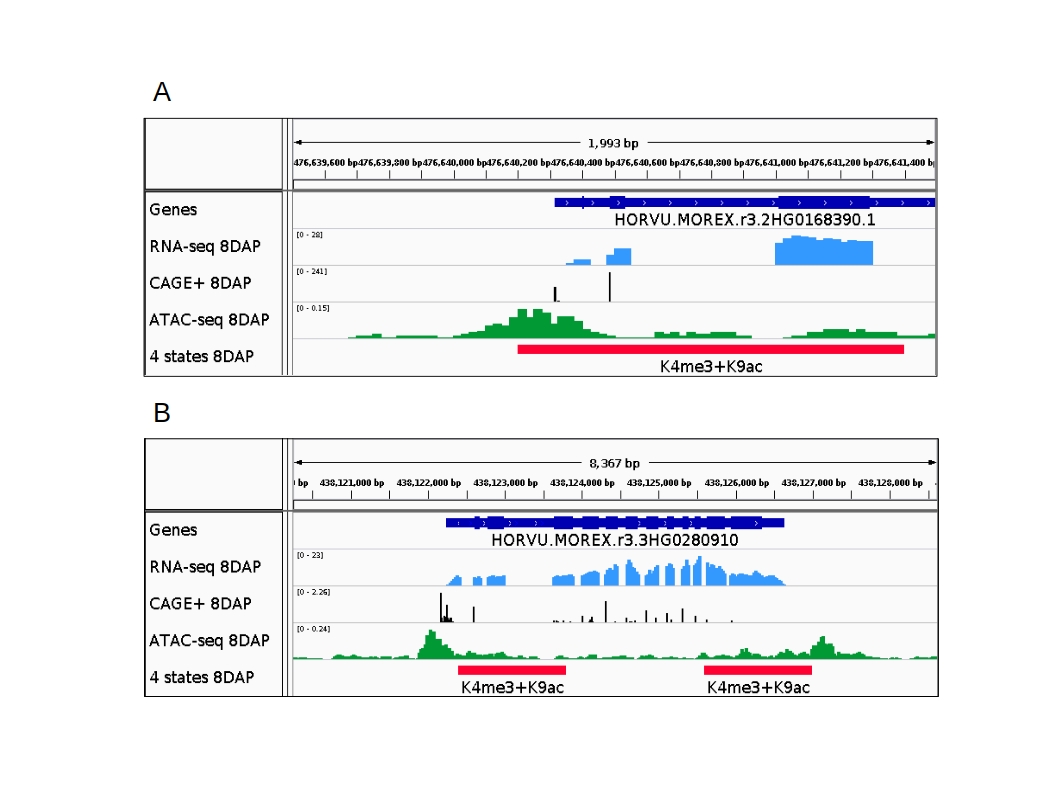


**Figure S4. Examples of secondary CTSSs. A)** Gene coding for 40S ribosomal protein S10 with a secondary TC (cluster 2) that originates from the first-intron splice acceptor. **B)** Example of a gene with multiple secondary TCs belonging to cluster sets 1-3 and 4-5.


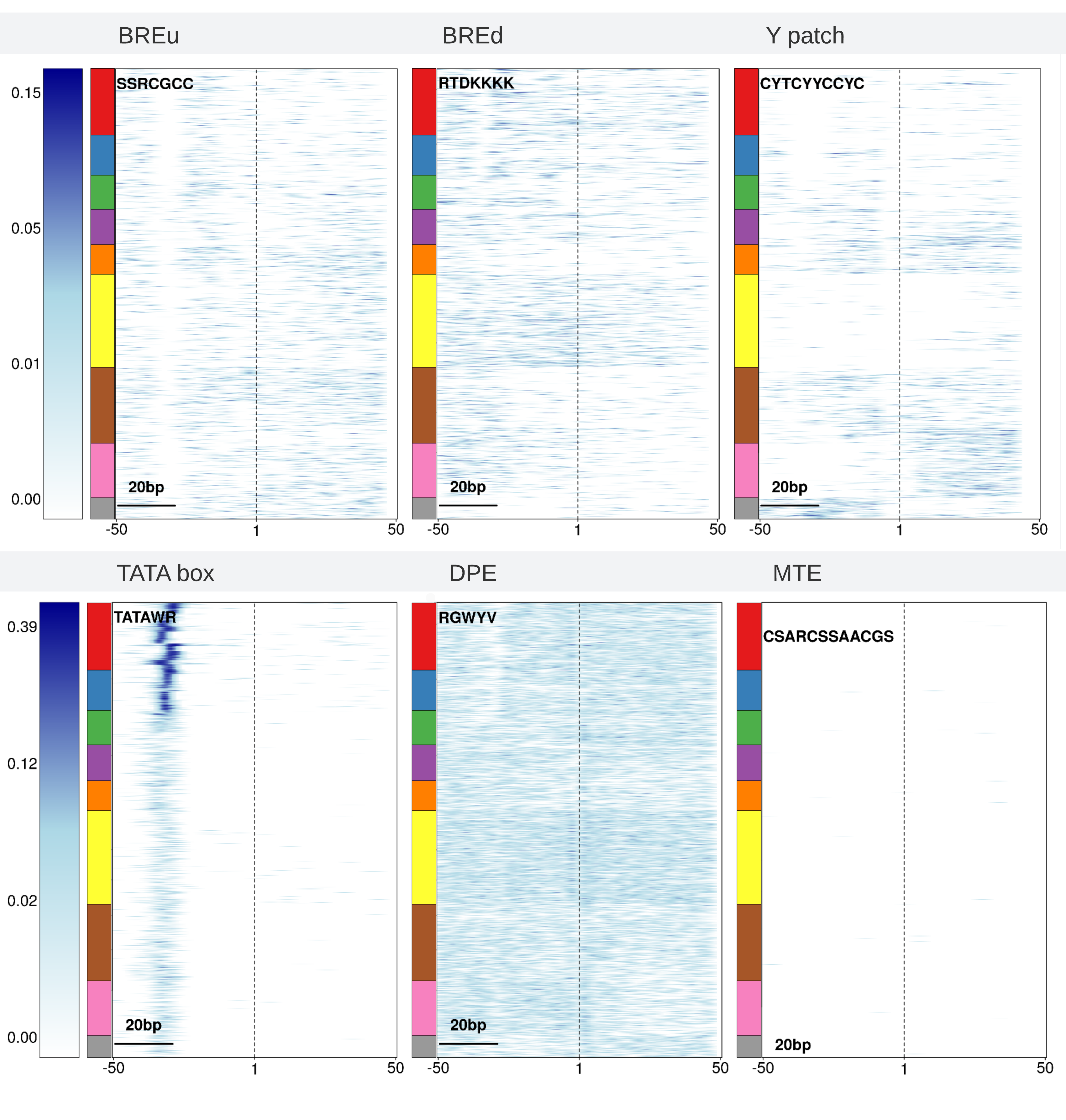


**Figure S5. Heat maps for known core promoter sequence motifs in the primary consensus promoter clusters.** The multi-coloured bars left of the heat maps indicate boundaries between the clusters, ordered as in Figure 2A. The heatmaps show enrichment of the given motif in the sequence with values 0-1.





**Figure S6. Clustering of +/- 100 bp CAGE promoter sequence architectures.** Stage-specific primary promoters were clustered using the SeqArchR algorithm and ordered by median interquantile widths (IQWs). The composed plots for **A**) 8DAP,  **B**) 24 DAP, **C**) 4DAG contain boxplots for IQW and gene expression level values (tags per million (TPM), log-transformed) per each cluster followed up by sequence logos and genomic feature annotation barplot.



**F****igure S7. Tissue specificity of individual stage-specific promoter clusters.** The tissue specificity is expressed as a tau value, which was calculated from barley RNA-seq data from multiple tissues [40]. A higher tau value indicates higher tissue specificity. Cluster designations are as in Figure S3. Note the higher tissue specificity of TATA-box promoter clusters (purple and blue colours).





C


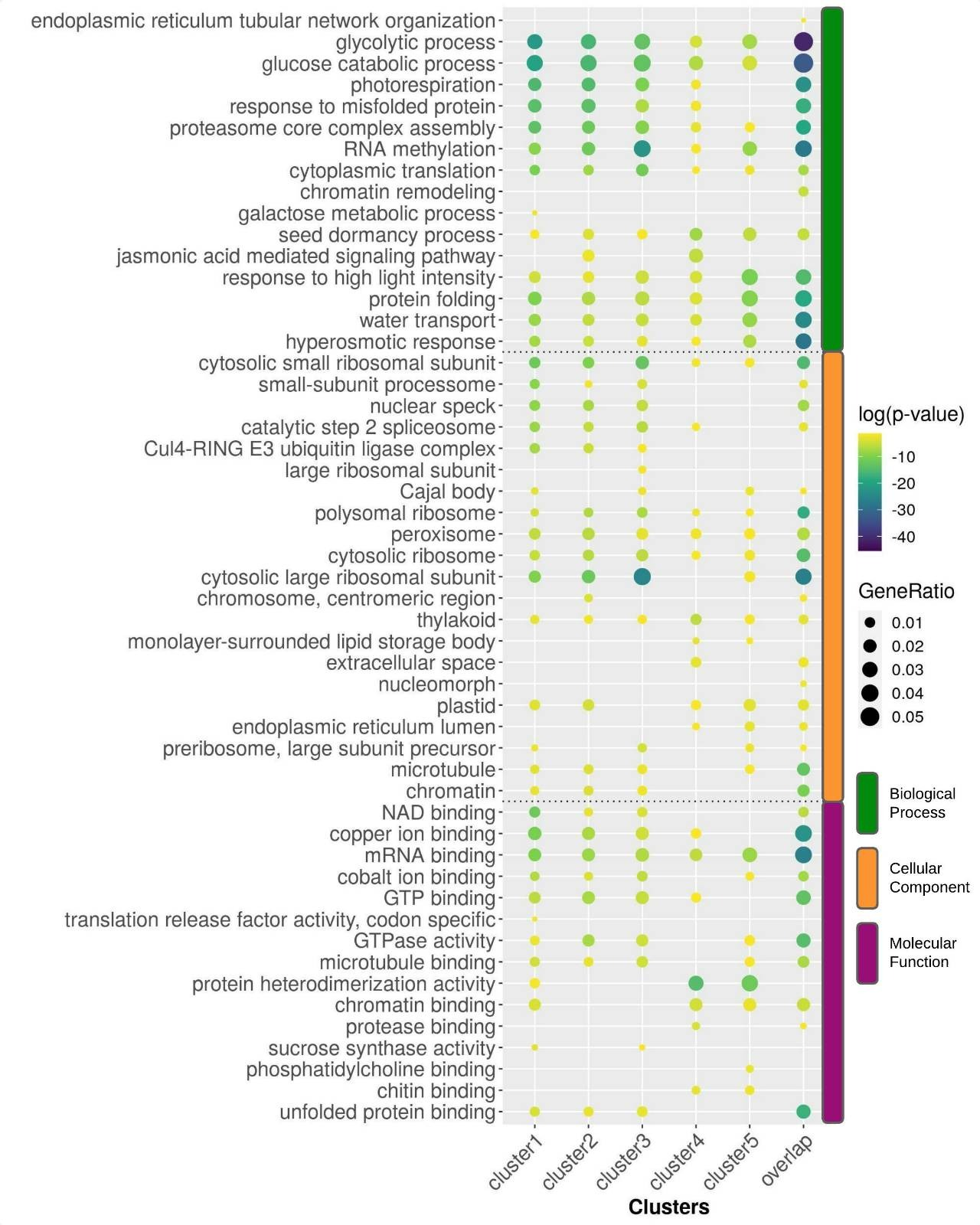


**Figure S8. Comparison of the published and the newly generated Morex GO term annotation and GO enrichment analysis for multi-secondary-TSS genes.** Top five enriched GO terms as in the published MorexV3 annotation [30] generated by AHRD pipeline **A**), and in the new Morex annotation generated by GOMAP toolkit **B**). The GOMAP resulted in better-defined gene categories. **C)** GO enrichment analysis of secondary clusters with a separated group of multi-TSS genes, assigned by SeqArchR to both cluster sets 1-3 and 4-5 ("overlap").


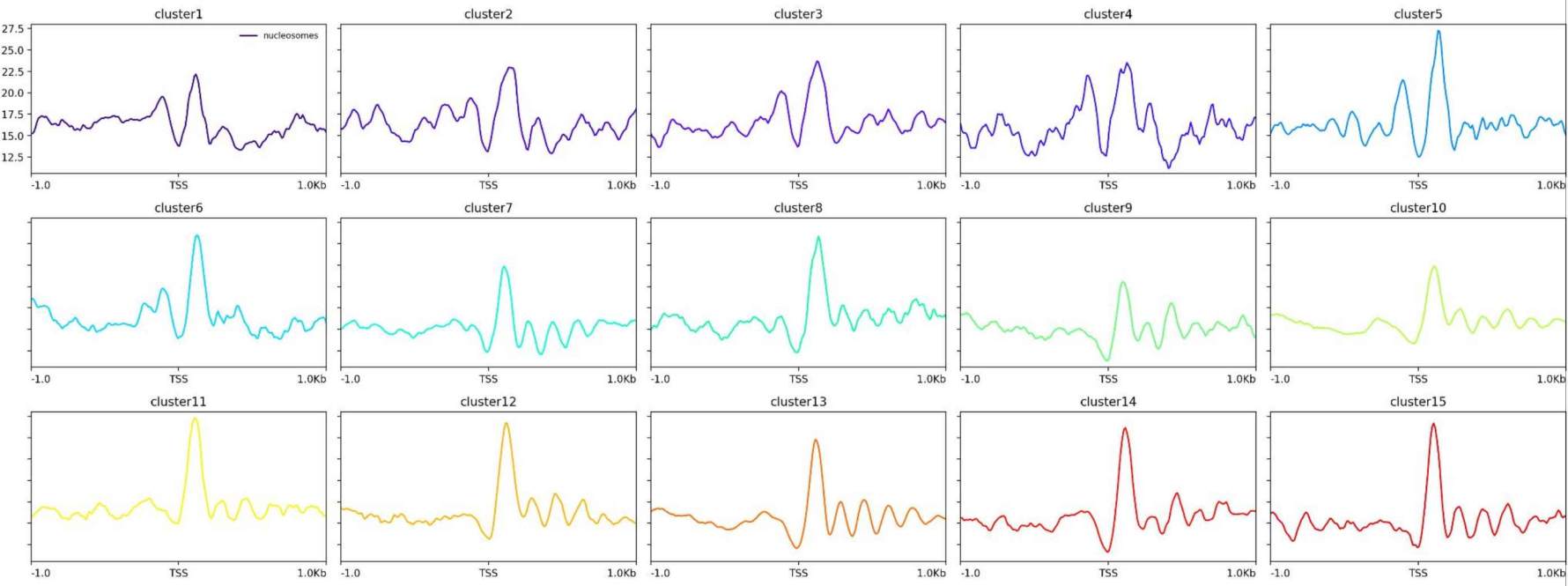


**Figure S9. Nucleosome positioning profiles +/-1000 bp around dominant CTSSs in 24DAP embryo.** The data show a phased nucleosome upstream TSS present in TATA-box promoters (clusters 1-6) only.



**F****igure S10. Chromatin profiles and TE promoter occupancy.** (**A**, **B**) Histone post-translational modification profiles in two representative 24DAP clusters. The heatmap indicates by color the scores for normalized counts in windows of -1000/+2000 bp around the TSS: **A**) TATA-box cluster 1 and **B**) non-TATA cluster 8, showing the difference in the H3K27me3 marking as a result of histone modification profiles grouped by k-means clustering. **C**) Promoter overlap with barley transposable elements.


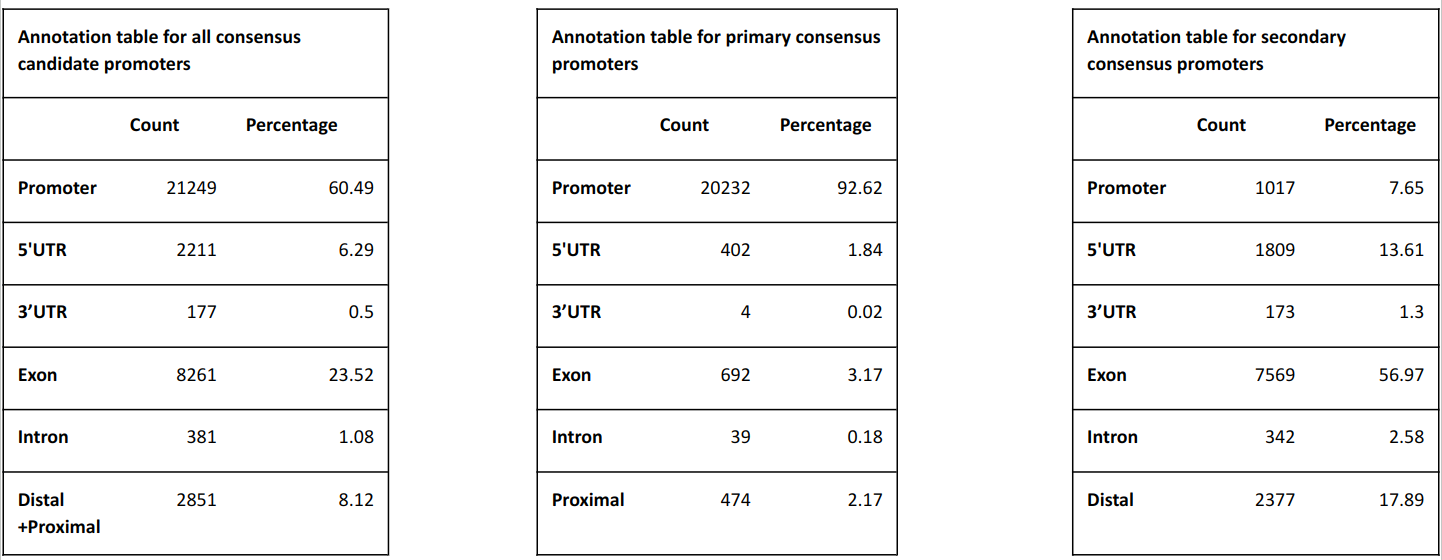


**Table S1: Annotation of consensus promoters.** The table corresponds to Figure 1c and contains count values for each annotation category.


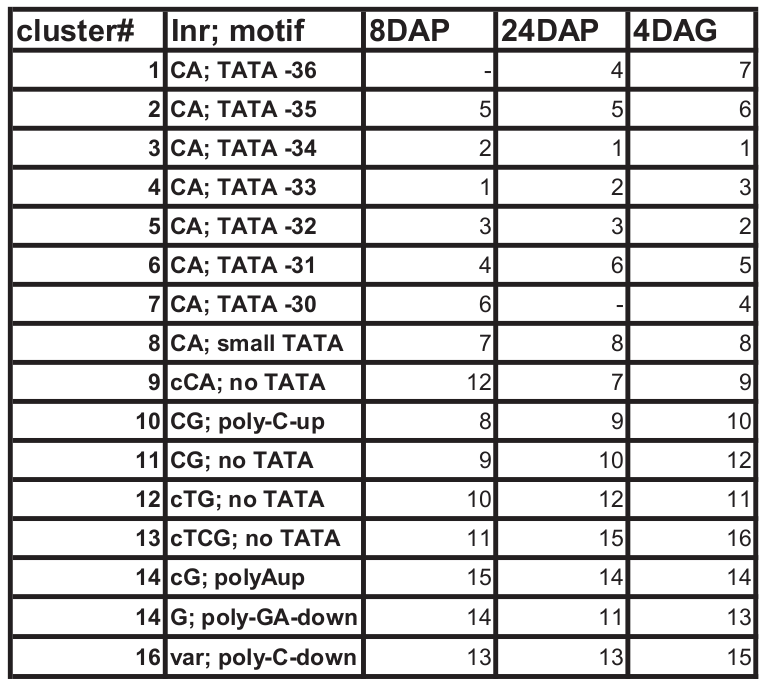


**Table S2. Relation of promoter clusters generated for three individual stages of embryo development** **to the consensus clusters**.


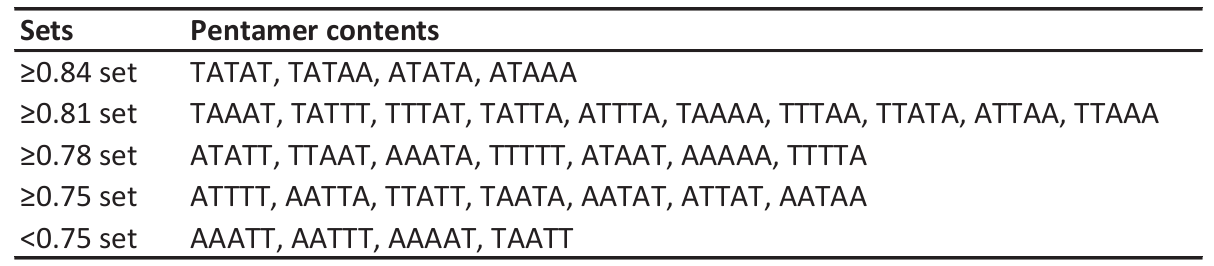


**Table S3: TATA-box-like motif sets** according to the level of correlation with the TATA-box PWM.
