# Supplementary material for: Core promoterome of barley embryo
Source: Comput Struct Biotechnol J. 2023 Dec 5;23:264–77. doi: 10.1016/j.csbj.2023.12.003 (PMC10762323; doi:10.1016/j.csbj.2023.12.003)
Supplement: Supplementary file 3 — Supplementary material. [file mmc3.docx]

**Table S1.**The annotation of consensus promoters corresponding to Figure 1C and containing count values for each annotation category.

| **Annotation table for all consensus candidate promoters** | | |  |  | **Annotation table for primary consensus promoters** | | |  |  | **Annotation table for secondary consensus promoters** | | |
| --- | --- | --- | --- | --- | --- | --- | --- | --- | --- | --- | --- | --- |
|  | **Count** | **Percentage** |  |  |  | **Count** | **Percentage** |  |  |  | **Count** | **Percentage** |
| **Promoter** | 21249 | 60.49 |  |  | **Promoter** | 20232 | 92.62 |  |  | **Promoter** | 1017 | 7.65 |
| **5'UTR** | 2211 | 6.29 |  |  | **5'UTR** | 402 | 1.84 |  |  | **5'UTR** | 1809 | 13.61 |
| **3’UTR** | 177 | 0.5 |  |  | **3’UTR** | 4 | 0.02 |  |  | **3’UTR** | 173 | 1.3 |
| **Exon** | 8261 | 23.52 |  |  | **Exon** | 692 | 3.17 |  |  | **Exon** | 7569 | 56.97 |
| **Intron** | 381 | 1.08 |  |  | **Intron** | 39 | 0.18 |  |  | **Intron** | 342 | 2.58 |
| **Distal**  **+Proximal** | 2851 | 8.12 |  |  | **Proximal** | 474 | 2.17 |  |  | **Distal** | 2377 | 17.89 |
